# Post‐translational modifications linked to preclinical Alzheimer's disease–related pathological and cognitive changes

**DOI:** 10.1002/alz.13576

**Published:** 2023-12-25

**Authors:** Olamide Abiose, Jarod Rutledge, Patricia Moran‐Losada, Michael E. Belloy, Edward N. Wilson, Zihuai He, Alexandra N. Trelle, Divya Channappa, America Romero, Jennifer Park, Maya V. Yutsis, Sharon J. Sha, Katrin I. Andreasson, Kathleen L. Poston, Victor W. Henderson, Anthony D. Wagner, Tony Wyss‐Coray, Elizabeth C. Mormino

**Affiliations:** ^1^ Department of Neurology and Neurological Sciences Stanford University School of Medicine Palo Alto California USA; ^2^ Wu Tsai Neurosciences Institute Stanford University School of Medicine Stanford California USA; ^3^ The Phil and Penny Knight Initiative for Brain Resilience Stanford University Stanford California USA; ^4^ Department of Genetics Stanford University Stanford California USA; ^5^ Center for Biomedical Informatics Research Stanford University School of Medicine Stanford California USA; ^6^ Chan Zuckerberg Biohub San Francisco California USA; ^7^ Department of Epidemiology & Population Health Stanford University School of Medicine Stanford California USA; ^8^ Department of Psychology Stanford University Stanford California USA

**Keywords:** aging, Alzheimer's disease, autophagy, cerebral spinal fluid, clinically unimpaired, protein co‐expression network, ubiquitination

## Abstract

**INTRODUCTION:**

In this study, we leverage proteomic techniques to identify communities of proteins underlying Alzheimer's disease (AD) risk among clinically unimpaired (CU) older adults.

**METHODS:**

We constructed a protein co‐expression network using 3869 cerebrospinal fluid (CSF) proteins quantified by SomaLogic, Inc., in a cohort of participants along the AD clinical spectrum. We then replicated this network in an independent cohort of CU older adults and related these modules to clinically‐relevant outcomes.

**RESULTS:**

We discovered modules enriched for phosphorylation and ubiquitination that were associated with abnormal amyloid status, as well as p‐tau_181_ (M4: *β* = 2.44, *p* < 0.001, M7: *β* = 2.57, *p* < 0.001) and executive function performance (M4: *β* = −2.00, *p* = 0.005, M7: *β* = −2.39, *p* < 0.001).

**DISCUSSION:**

In leveraging CSF proteomic data from individuals spanning the clinical spectrum of AD, we highlight the importance of post‐translational modifications for early cognitive and pathological changes.

## 1 BACKGROUND

Alzheimer's disease (AD) is the most common form of dementia among adults 65 years and older.^1^ The hallmark pathological features of AD are extracellular deposits of the misfolded amyloid beta (Aβ) protein, as well as neurofibrillary tangles composed of hyperphosphorylated tau protein.^2^ Protein misfolding causes soluble versions of Aβ and tau to be organized into toxic, fibrillar aggregates and to lose their functional properties.^3^, ^4^ As a result, there is growing interest in targeted, therapeutic interventions that enhance the biological processes behind protein degradation as a means of combating disease progression.^5^

The presence of amyloid plaques begins years if not decades before the onset of clinical dementia, and has been conceptualized as an asymptomatic preclinical stage of AD.^6^, ^7^ Abnormal Aβ accumulation among clinically unimpaired (CU) adults is associated with magnetic resonance imaging (MRI)–based measures of neuronal injury,^8^, ^9^ abnormal tau levels,^10^ cognitive decline,^11^, ^12^ and future progression to mild cognitive impairment (MCI) or dementia.^11^ Decreases in cerebrospinal fluid (CSF) levels of Aβ_42_ and Aβ_42_ /Aβ_40_ ratios are among the earliest physiological changes that can be used to identify individuals with preclinical AD.^13^, ^14^ This initial period along the AD continuum represents a promising target for early therapeutic intervention^15^; however, the physiological drivers of initial disease processes remain poorly understood.

Recent advancements in mass spectrometry, immunoaffinity assays, and aptamer‐based microarrays have led to recent findings describing proteomic changes beyond amyloid and tau in the context of AD.^16^, ^17^ This research has been pivotal to identifying novel biomarkers and disease processes that emerge in parallel to or independent of initial amyloid and tau accumulation. As opposed to focusing solely on individual protein levels, many of these studies have approached high throughput proteomic data sets using network approaches to uncover communities of proteins—or modules—important to AD pathogenesis.

The Accelerated Medicines Partnership for Alzheimer's Disease (AMP‐AD) Consortium alone has published nearly a dozen such studies investigating proteomic changes in AD,^18^, ^19^, ^20^, ^21^, ^22^, ^23^, ^24^, ^25^, ^26^ often exploring changes that occur in the asymptomatic period preceding AD dementia, that is, among CU individuals and individuals with MCI who do not meet clinical criteria for dementia.^18^, ^21^, ^22^, ^23^, ^24^, ^25^, ^26^ These studies^6^, ^22^, ^23^, ^25^, ^27^ have identified proteomic changes in participants without dementia (defined as having a Mini‐Mental State Exam [MMSE] score above 24 or Clinical Dementia Rating [CDR] score less than 1^23^).

However, few studies—including those described above—have utilized network techniques to examine proteomic changes associated with preclinical AD, particularly among CU individuals.^28^ Abnormal amyloid accumulation during the CU stage is increasingly becoming a target for therapeutic intervention in anti‐amyloid clinical trials, yet little is known about the biological pathways underlying in vivo neurodegenerative and cognitive changes at the earliest stages of the AD cascade, among CU individuals.

In this study, we identify CSF proteomic co‐expression modules and examine their associations with early disease‐relevant changes in a large cohort of CU individuals. To accomplish this, we constructed a protein co‐expression network among a discovery cohort of CU, MCI, and AD participants, using 3869 proteins quantified in the CSF by modified aptamer technology (SomaScan). We further replicated this network in an independent, deeply phenotyped CU cohort. We discovered modules enriched for post‐translational modifications (phosphorylation and ubiquitination) that predicted abnormal amyloid accumulation, tau aggregation, cognitive performance, and apolipoprotein E (*APOE*) genotype among CU individuals. These findings emphasize the importance and multi‐faceted role of post‐translational modifications as an early driver of AD‐related pathophysiology.

## 2 METHODS

### 2.1 Participants

We analyzed CSF samples from 258 research participants recruited from either the Iqbal Farrukh and Asad Jamal Stanford Alzheimer's Disease Research Center (ADRC) and its affiliated clinics (ADRC+) or from the Stanford Aging and Memory Study (SAMS). Clinical diagnosis was determined at a clinical consensus meeting by a panel of neurologists and neuropsychologists. ADRC+ participants underwent neurological examination, neuropsychological testing, and neuroimaging, and provided biofluid samples (CSF). Participants diagnosed as CU (CDR = 0 or 0.5), MCI (CDR = 0, 0.5, or 1), or having AD dementia (CDR > 0.5) were used in our analyses and treated as the discovery cohort.^29^ SAMS is an ongoing prospective study of CU older adults that seeks to understand how memory performance relates to brain structure, brain function, and AD risk factors.^30^, ^31^ SAMS eligibility included normal or corrected‐to‐normal vision/hearing, right handedness, native English speaking, a lack of a history of neurologic or psychiatric disease, a CDR global score of zero, and performance within the normal range on a standardized neuropsychological test battery. SAMS participants underwent lumbar puncture to collect CSF and completed MRI scanning.

All study protocols were approved by the Stanford University Institutional Review Board. Written informed consent was obtained from each study participant or their legally authorized representative.

**RESEARCH IN CONTEXT**

1. **Systematic review**: We reviewed the literature using traditional methods (e.g., Google Scholar) for studies using network approaches to analyze proteomic changes among clinically unimpaired (CU) older adults at increased risk for Alzheimer's disease (AD) (i.e., with abnormal levels of amyloid beta [Aβ] aggregation). The research we found in this area either had small sample sizes (N < 20) or grouped CU participants with those meeting the criteria for mild cognitive impairment (MCI).
2. **Interpretation**: In this study, we leverage cerebrospinal fluid (CSF) data and proteomic techniques—particularly co‐expression networks—to identify biological mechanisms related to AD pathology among CU older adults. Our results highlighted the involvement of ubiquitination, particularly as a regulator of autophagy, in such early disease pathology.
3. **Future directions**: Future research should determine the causal direction between autophagy and abnormal amyloid accumulation, as well as examine proteomic changes among CU individuals longitudinally and among more diverse participant samples.

### 2.2 Cerebrospinal fluid samples

CSF samples were collected via lumbar puncture, which was performed in the morning after an overnight fast. A Sprotte needle inserted between lumbar vertebrae L4 and L5 was used to collect 10 mL of CSF, divided into 1.0 or 0.5 mL aliquots and stored in polypropylene tubes at −80°C until assay. CSF centrifugation and assessment of blood contamination was conducted as described previously.^32^

### 2.3 AD biomarker quantification and amyloid status determination

Separate aliquots processed by the Lumipulse G system (Fujirebio US, Inc., Malvern, PA) were used to measure CSF levels of AD biomarkers (phosphorylated tau 181 [p‐tau_181_], Aβ_42_, and Aβ_40_) for all 147 SAMS CU and 89 of the 111 ADRC+ participants.^29^ The remaining 22 ADRC+ participants who did not have Lumipulse data had Aβ peptides quantified by the Quanterix Neurology 3‐plex A assay (Quanterix, MA, USA). Amyloid status was determined with ratios of Aβ_42_ to Aβ_40_, and Aβ_42_/Aβ_40_ ratios were used both continuously and dichotomously in subsequent analyses. Cut offs to classify participants into amyloid negative (Aβ−) and amyloid positive (Aβ+) groups were derived in a batch‐specific fashion and are described in the Supplementary Methods.

### 2.4 SomaLogic protein quantification and quality control

The aptamer‐based SOMAScan assay platform was used to quantify CSF protein expression levels for further network analysis.^33^ This method of protein quantification relies on chemically modified DNA strands whose unique three‐dimensional (3D) shapes allow them to bind to specific proteins with high specificity. “SOMAmers” whose protein‐aptamer complexes that survive sequential streptavidin bead capture, photocleavage, and kinetic capture are quantified after hybridizing to a DNA microarray. This technique provided us with the relative concentration (quantified in terms of relative fluorescent units, or RFUs) of 5284 CSF proteins.

SomaLogic, Inc., uses a 96‐well plate design with wells devoted to buffer, calibrator, quality control, and biological samples to account for nuisance variation and batch effects. There are three stages of data normalization: (1) *hybridization control normalization* removes individual sample variance by using hybridization control spike‐ins to calculate a factor by which to scale each sample's measurements; (2) *median signal normalization* accounts for intraplate measurement variance within a sample class; and (3) *plate scaling and calibration* calculates within‐plate and across‐plate reference values based on control calibrator samples to adjust the intraplate measurements of each individual protein, as well as the entire plate as a whole. Proteins flagged by SomaLogic's internal quality control, as well as samples with normalization factors falling outside the acceptable assay range were removed before analysis. In addition, we removed outlying samples whose standardized connectivity^34^ was more than three standard deviations (SD) from the mean. Finally, for each protein, we constructed a distribution of measurements across buffer samples and assessed whether it differed significantly from each clinical sample's measurement (at a false discovery rate alpha level of 5%). We removed proteins where more than 25% of clinical samples fell within this buffer distribution, resulting in 3869 CSF proteins for subsequent analyses (Figure S1).

### 2.5 MRI imaging

MRI was used to measure structural neuroimaging outcomes within the SAMS CU cohort. Data were acquired on a 3T GE Discovery MR750 MRI scanner (GE Healthcare) using a 32‐channel radiofrequency receive‐only head coil (Nova Medical). For the current analyses, we processed a whole‐brain high‐resolution T1‐weighted anatomic volume (repetition time [TR] = 7.26 ms, field of view [FoV] = 230 mm × 230 mm, voxel size = 0.9 × 0.9 × 0.9 mm, slices = 186), through FreeSurfer version 7. Subcortical and cortical region of interest (ROI) volumes—including total gray matter, hippocampus, and white matter hypointensity volume—were defined by FreeSurfer's aparc+aseg atlas.

### 2.6 Cognitive composite scores

We examined memory and executive function composite scores derived from a neuropsychological battery administered to the SAMS CU cohort. The memory composite score reflected delayed recall performance across (1) the logical memory subtest of the Wechsler Memory Scale, (2) the Hopkins Verbal Learning Test—Revised, and (3) the Brief Visuospatial Memory Test—Revised. The executive function composite score was derived by averaging the (1) total time to complete Trails B, (2) total number of animals recalled in 60 s, and (3) the summed score from the Digit Span Forward and Backward. Trails B was inverted such that higher scores reflect better performance. Composite scores were computed by first z‐scoring individual subtest scores using the full SAMS cohort as reference and then averaging.^31^

### 2.7 *APOE* genotyping

The *APOE* genotype was determined by whole‐genome sequencing (WGS) at either the Beijing Genomics Institute (BGI) in Shenzhen, China, or as part of the Stanford Extreme Phenotypes in Alzheimer's Disease project with sequencing performed at the Uniformed Services University of the Health Sciences (USUHS) on an Illumina HiSeq platform. The Genome Analysis Toolkit (GATK) workflow Germline short variant discovery was used to map genome sequencing data to the reference genome (GRCh38) and to produce high‐confidence variant calls using joint‐calling.^35^ *APOE* genotype (ε2/ε3/ε4) was determined using allelic combinations of single nucleotide variants rs7412 and rs429358.

### 2.8 Statistical methods

All statistical analyses were performed with R version 4.2.2. Network construction, module stability/preservation, and differential expression analyses were conducted with 111 participants recruited from the ADRC and affiliated clinics (69 CU, 22 with MCI, and 20 with AD dementia), after using hierarchical clustering to remove eight outlying participants. Proteomic data were log_10_ transformed and adjusted for effects of age, sex, length of CSF storage time, study origin (i.e., either ADRC, SAMS, or affiliated clinics), and hidden factors identified by the first five components of singular value decomposition. As a final quality control step, we conducted a principal components analysis on the entire proteomic data set and found no lingering batch effects (Figure S1).

### 2.9 Differential abundance analyses

We conducted a one‐way analysis of variance (ANOVA) followed by a Student's *t*‐test to identify differentially expressed proteins in AD dementia compared to CU individuals within the ADRC+ cohort. A false discovery rate (FDR) correction at an alpha level of 5% was used to account for multiple comparisons and determine significance. Modules with at least one differentially abundant protein were considered relevant to aging and AD dementia, and they became the focus of subsequent analyses.

### 2.10 Protein–protein co‐expression network

We performed a weighted gene correlation network analysis using the WGCNA package (version 1.72.1) in R.^34^ For this, we used a subset of participants from the ADRC+ cohort to ensure a diagnostically balanced sample; specifically, we included only 18 CU, 18 MCI, and 18 AD participants. All cognitively impaired participants (MCI and AD) were amyloid positive.

First, we constructed a matrix of the bi‐weight mid‐correlations between proteins and transformed this into a signed adjacency matrix using a soft thresholding power of 12 (resulting in a scale‐free topology fit above 0.8). This adjacency matrix was then transformed into a topological overlap matrix (TOM), which captures the similarity between nodes in terms of their shared patterns of connections. We performed hierarchical clustering with a 1‐TOM distance measure, and used a dynamic tree cutting algorithm (cutreeDynamic, with a minimum module size of 15, deepSplit = 4, and a partitioning around medoid step that respected the dendrogram) to identify modules from the dendrogram.

The first principal component of each module's protein expression matrix was used to define a module eigenprotein.^34^ The degree of module membership for each protein (i.e., their intramodular connectivity (kME) value) is calculated by correlating its expression patterns across all samples with the module eigenprotein. We used kME values to merge highly similar modules together. The top 50% of proteins (ranked by kME value) within each module were correlated with every other module; if more than 25% of these proteins had greater membership in another module, the modules were merged.

### 2.11 Gene ontology analysis

We used the g:Profiler R package (version 0.2.1) to understand the Gene Ontology (GO) biological processes (GO:BP) and molecular functions (GO:MF) enriched within our modules at an experiment‐wide threshold of α = 0.05. Whenever possible, we used the program default multiple comparison algorithm (g:SCS), which accounts for the hierarchical relationship between GO terms; we also tested for enrichment against a custom background of all 5284 SomaLogic‐quantified proteins. When we were unable to find significantly enriched biological pathways with this approach, we turned to three alternate methods. First, we used FDR correction for multiple comparisons (still enriched against the custom background of all SomaLogic proteins). If we were still unable to identify significant GO terms, we then used g:SCS correction against a background of all annotated genes. Finally, if we were still unable to identify GO terms, we used FDR correction against a background of all annotated genes.

### 2.12 Module preservation analysis

We used the WGCNA modulePreservation function to calculate the extent to which our modules were preserved in the independent, CU cohort (SAMS). This function applied our previously‐defined modules to SAMS CSF samples and calculated module preservation statistics comparing the strength of interrelationship between nodes (module density) as well as connectivity patterns (module connectivity) in replicated modules versus the original.^36^ For each preservation statistic, module labels were permuted 200 times with a random seed set to 1 for reproducibility. Module density and connectivity preservation statistics are captured in a Z *_Summary_* measure (i.e., the mean of these two categories of preservation statistics); modules with a Z *_Summary_* >10 were considered preserved, as recommended previously.^36^ We additionally examined the *medianRank* of each module, which reflects the relative ranking of each across all preservation statistics and is less influenced by module size than the Z *_Summary_* value.^36^

### 2.13 Module/phenotype relationships

Representative eigenproteins were used to capture protein‐expression patterns within each module, and to conduct statistical analyses examining module/trait associations. Kruskal–Wallis tests for one‐way ANOVA were used to calculate module relationships to clinical diagnosis within the ADRC+ cohort and Aβ status within the SAMS CU cohort. All module associations with aging and AD‐related phenotypes—Aβ_42_/Aβ_40_ ratios, log‐transformed p‐tau_181_ levels, hippocampal and total gray matter volume, log‐transformed white matter hypointensities, *APOE* ε2 and ε4 allele count, and memory and executive function cognitive composite scores—were determined by linear regression models controlling for age and sex. Models predicting cognition additionally controlled for education level, and those predicting MRI outcomes further controlled for estimated intracranial volume. These demographically adjusted models comprised our first set of analyses (approach 1). In a second round of analysis (approach 2), we additionally controlled for amyloid status (and removed CSF Aβ_42_/Aβ_40_ ratios as an outcome of interest). Finally, we performed a set of analyses (approach 3) that controlled both for amyloid status and continuous CSF p‐tau_181_ levels (removing CSF Aβ_42_/Aβ_40_ ratios and log‐transformed CSF p‐tau_181_ levels as outcomes of interest). All *p*‐values were adjusted for multiple comparisons at an FDR significance level of *p* < 0.05.

### 2.14 Module enrichment analyses

To establish whether a module was enriched with a particular characteristic—such as genetic regulators of amyloid pathology or proteins differentially abundant in AD dementia versus CU individuals—we first calculated the average log‐transformed *p*‐value for that given characteristic across proteins within our module of interest. We then constructed a null distribution of average *p*‐values with 10,000 module‐sized random samples (with replacement) and calculated a z score to see if there was a significant difference between our module of interest compared to the null distribution.

To assess for enrichment of amyloid pathology, we used a single‐nucleotide polymorphism (SNP) summary statistics from the genome‐wide association study (GWAS) of amyloid positron emission tomography (PET) data of Raghavan et al. (2020).^37^ For enrichment of genetic regulators of clinical AD dementia diagnosis, we used GWAS summary statistics from the International Genomics of Alzheimer's Project (https://www.niagads.org/igap‐rv‐summary‐stats‐kunkle‐p‐value‐data),^38^ as well as the study of Bellenguez et al. (2022) (https://www.ebi.ac.uk/gwas/publications/35379992).^39^ GWAS summary statistics served as input to the FUMA online platform, which functionally annotates SNPs, maps them onto genes, and calculates the gene‐level associations with a given phenotype.^40^ The *p*‐values resulting from these gene‐level associations were used for our module enrichment analyses of amyloid PET signal and AD dementia genetic risk.

To assess whether a given module was enriched for polyubiquitinated proteins, we used a mapping of the ubiquitylome by Abreha et al. (2018),^41^ and to examine enrichment for protein phosphopeptides, we used a mapping of the phosphoproteome by Ping et al. (2020).^42^ For these analyses, instead of calculating the average log‐transformed *p*‐values, we calculated the average number of ubiquitination sites or protein phosphopeptides within a module of interest or an equally‐sized random sample.

We performed cell‐type enrichment analyses using the Internet‐based application, WebCSEA (https://bioinfo.uth.edu/webcsea/index.php?csrt=11311320302846866589). Modules were considered enriched for a particular cell type if the combined *p*‐value exceeded a Bonferroni threshold of (*p* = 3.69 × 10^−5^).

### 2.15 Multivariate LASSO regression, stability selection, and other statistical analyses

We performed multivariate regression analyses with a least absolute shrinkage and selection operator (LASSO) method to examine whether protein modules of interest could discriminate Aβ− from Aβ+ CU participants, using the glmnet package (version 4.1.6) in R. This approach uses L1 regularization to reduce the number of parameters within a model, by shrinking irrelevant and redundant parameters to a coefficient of 0. We selected the tuning parameter, λ, that minimized the mean cross‐validated error after 10‐fold cross‐validation. We also manually assigned observations to folds 1 through 10 using a random sequence. SAMS CU participants with *APOE* genotype information (*n* = 124) were divided into an 80/20 train/validation split and used to train and validate our classifiers. We then evaluated the performance of our classifiers among a test set of ADRC+ CU participants that were not included in our network construction process (*n* = 54). We used the mean and confidence interval of the area under the receiver‐operating characteristic (ROC) curve (AUC) to determine the significance and accuracy of each of our classifiers; these calculations and visualizations were performed with the ROCR (version 1.0.11) and pROC (1.18.0) R packages.

For the simplest logistic regression model, we included only age, sex, and ε4 allele count as predictors of amyloid status. We used this as a baseline point of comparison for our LASSO regression model, which additionally included all proteins within a given network module as predictors. The purpose of this was to leverage the variable selection properties of LASSO regression to understand which module proteins and/or demographic characteristics were most influential in predicting amyloid positivity. Thus we implemented a stability selection approach using the stabs R package (version 0.6.4), which uses subsampling to determine which model features are most likely to be selected across many different LASSO iterations.^43^, ^44^ Each subsample contained half of the observations of the original data set, and this process was repeated 50 times. We used a 65% selection probability threshold to identify stably selected LASSO regression model features; these features then served as predictors in a logistic regression model predicting amyloid status among CU individuals.

## 3 RESULTS

### 3.1 ADRC+ discovery cohort participant characteristics

We began with data from 111 participants along the AD continuum as our discovery cohort (mean age = 68.6 years, SD = 8.31; 55% women) (Table 1).

**TABLE 1:** Demographic information for the Alzheimer's Disease Research Center and affiliated clinics (ADRC+ cohort).

	Full cohort, N = 111^1^	Network construction subset, N = 54^1^
Age, years	68.62 (8.31)	68.63 (8.32)
Gender		
Female	61 (55%)	33 (61%)
Male	50 (45%)	21 (39%)
Sample origin		
ADRC participant	78 (70%)	35 (65%)
Clinic non‐enrollee	33 (30%)	19 (35%)
Length of CSF storage time, years	3.94 (2.40)	4.24 (2.63)
Amyloid status		
Negative	42 (38%)	12 (22%)
Positive	69 (62%)	42 (78%)
Diagnosis		
CU	73 (66%)	18 (33%)
MCI	19 (17%)	18 (33%)
AD	19 (17%)	18 (33%)
*APOE* ε4 allele count		
0	35 (45%)	13 (37%)
1	31 (40%)	18 (51%)
2	12 (15%)	4 (11%)
Unknown	33	19

^1^ Mean standard deviation (SD); *n* (%).

These participants were either enrolled by the Stanford ADRC or recruited from associated clinics at Stanford; we thus refer to these participants as the ADRC+ cohort. Of these participants, 73 were CU, 19 had MCI, and 19 were diagnosed with AD dementia. Based on CSF analyses, there were 42 amyloid negative (Aβ−) and 69 amyloid positive (Aβ+) participants; all clinically impaired participants (diagnosed with either MCI or AD) were amyloid positive. The mean length of storage time of CSF samples before protein quantification by the SomaScan assay platform was 3.94 years (SD = 2.39).

We used the ADRC+ cohort to identify proteins differentially abundant in AD relative to CU contexts. However, a clinically balanced subset of these participants (*n* = 54) were used to construct a protein co‐expression network (mean age = 68.6, SD = 8.32; 61% women). This subset included 18 CU (Aβ+: *n* = 6), 18 MCI, and 18 AD participants (Table 1).

### 3.2 Protein co‐expression network construction and module characterization reveals 13 AD‐relevant modules

**FIGURE 1:**
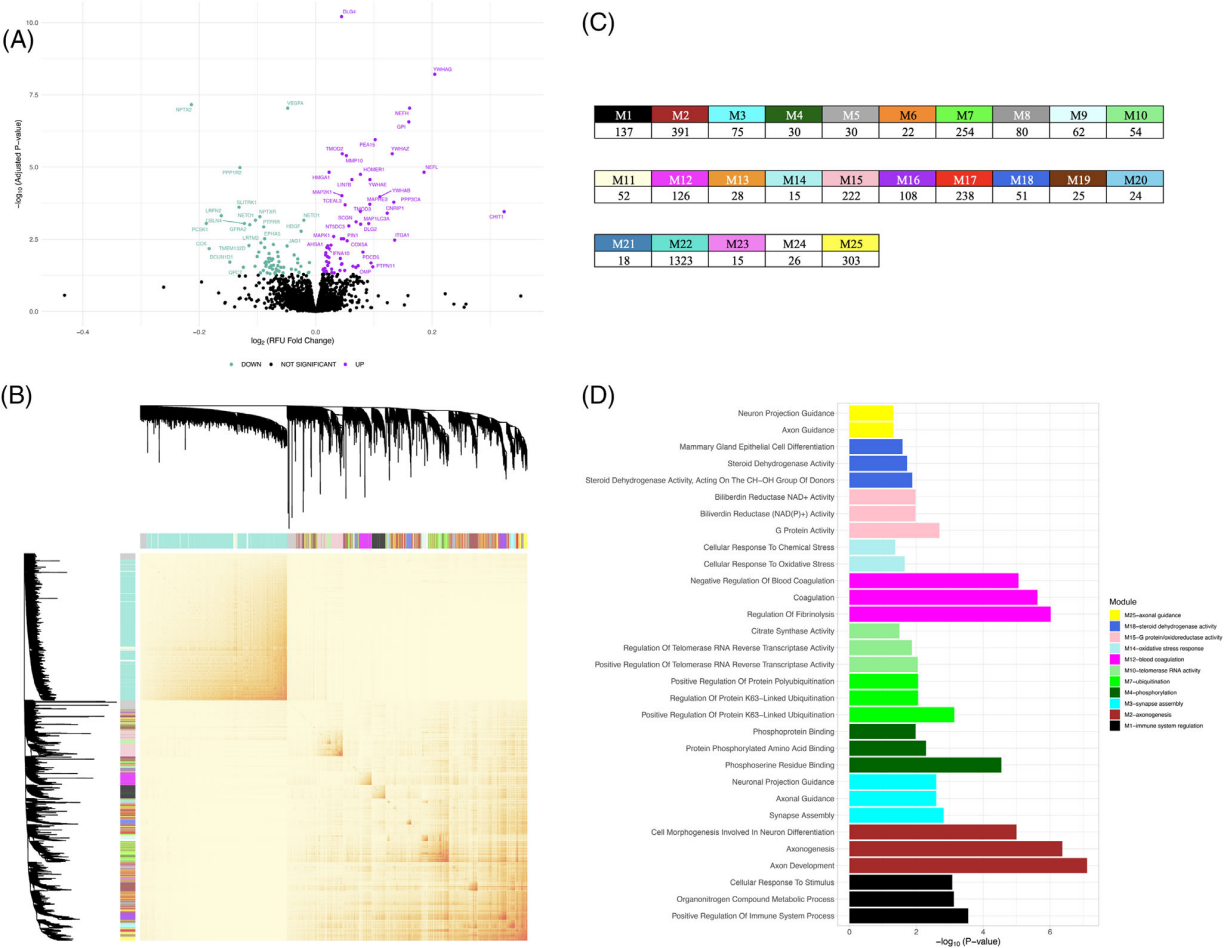
Protein co‐expression network construction and module characterization in the discovery ADRC+ cohort. (A) Differential abundance analysis. A volcano plot depicting the results of an ANOVA analysis followed by a Student's *t*‐test to identify differentially abundant proteins in AD dementia compared to CU individuals. This models the log2‐fold change in relative fluorescence units (RFUs) (x‐axis) against the negative log10 *p*‐value (y‐axis) representing the association between the protein and a clinical AD dementia diagnosis. The *p*‐values were adjusted using an FDR correction for multiple comparisons at an alpha level of 5%; only proteins with –log10 adjusted *p*‐values exceeding this threshold were colorized as teal (decreased abundance in AD) or purple (increased abundance in AD). These proteins were used to restrict the scope of our subsequent analyses of co‐expression network modules. (B) WGCNA protein co‐expression network construction. A heatmap representing the topological overlap matrix (TOM) based on similarities in protein abundance levels that was used as input for our hierarchical clustering and community detection. Heatmap colors range from light yellow to red, reflecting low to high similarity, respectively. At the top and to the right, the network dendrogram and module color assignments are displayed. (C) Table of module sizes. A table listing all modules in our ADRC+ network by the number of proteins within each module. (D) Gene ontology analysis. Functional annotations derived from gene ontology analyses of the modules containing at least one protein differentially abundant in AD, conducted using g:Profiler. Of these nine modules, only seven contained functional enrichments that exceeded significance thresholds, and they are depicted here. The top three most significant gene ontology (GO) biological process and/or molecular function terms per module are displayed (y‐axis) against their respective –log10 *p*‐values (x‐axis).

Differential expression analysis among the discovery ADRC+ cohort revealed 130 proteins whose CSF concentrations were significantly different between AD dementia and CU participants, at a FDR threshold of *p* < 0.05 (Figure 1A). These included AD‐associated proteins previously identified in the literature, such as vascular endothelial growth factor A (VEGFA, *p* = 9.159 × 10^−8^), matrix metalloproteinase 10 (MMP‐10, *p* = 4.045 × 10^−6^), and neurofilament light polypeptide (NEFL, *p* = 1.515 × 10^−5^).^45^, ^46^, ^47^, ^48^ A number of 14‐3‐3 regulatory proteins were also differentially expressed between healthy and AD dementia participants, including YWHAB (*p* = 1.066 × 10^−4^), YWHAE (*p* = 2.729 × 10^−5^), YWHAG (*p* = 6.159 × 10^−9^), and YWHAZ (*p* = 3.477 × 10^−6^).

We constructed a protein co‐expression network among a clinically balanced subset from the ADRC+ cohort, using the weighted gene correlation network analysis (WGCNA) algorithm. This network resulted in 25 communities of proteins, or “modules,” ranging in size from 15 (M14‐oxidative stress response and M23) to 1323 proteins (M22) (Figure 1B,C). Thirteen of these modules were enriched with at least one of the 130 differentially expressed proteins. We considered these modules to be AD relevant and made them the focus of subsequent analysis interpretations.

Gene ontology (GO) analyses allowed us to characterize the biological processes and molecular functions enriched in 23 of the 25 modules, including 11 of the 13 AD‐relevant modules (Figure 1D, Figure S2). These modules and annotations include M1‐immune system regulation, M2‐axonogenesis, M3‐synapse assembly, M4‐phosphorylation, M7‐ubiquitination, M10‐telomerase RNA activity, M12‐blood coagulation, M14‐oxidative stress response, M15‐G protein/oxidoreductase activity, M18‐steroid dehydrogenase activity, M22, M23, and M25‐axonal guidance.

**FIGURE 2:**
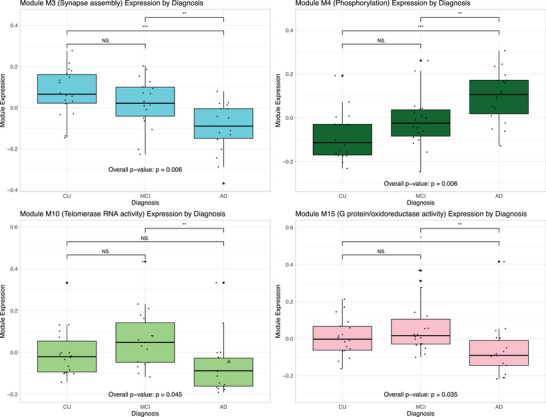
Modules by clinical disease stage. Box plots illustrating the results of Kruskall–Wallis tests for one‐way ANOVA used to calculate module eigenprotein relationships to clinical disease stage. Modules M3‐synapse assembly, M4‐phosphorylation, M10‐telomerase RNA activity, and M15‐G protein/oxidoreductase activity were significantly associated with disease stage after FDR correction for multiple corrections.

A representative eigenprotein was calculated for each module and used in Kruskall–Wallis tests to predict clinical disease stage (Figure 2). Four modules were significantly associated with AD severity after FDR correction for multiple comparisons, including M3‐synapse assembly (*p* = 0.0059), M4‐phosphorylation (*p* = 0.0059), M10‐telomerase RNA activity (*p* = 0.0447), and M15‐G protein/oxidoreductase activity (*p* = 0.0349). Modules M2‐axonogenesis (*p* = 0.0351) and M18‐steroid dehydrogenase activity (*p* = 0.0539) had weak to suggestive associations with clinical disease stage before multiple comparison correction (Figure S3).

### 3.3 Module preservation within an independent CU cohort (SAMS)

An independent cohort of 147 CU participants were used to examine whether the protein co‐expression network was preserved in the absence of cognitive impairment, as well as to relate modules from the network to clinically‐relevant phenotypes (mean age = 68.7 years, SD = 5.79; 61% women) (Table 2). These participants were enrolled in the Stanford Aging and Memory Study (SAMS) and are referred to herein as the SAMS CU cohort. There were 109 amyloid negative (Aβ−; mean age = 68.1 years, SD = 5.52) and 38 amyloid positive (Aβ+; mean age = 70.2 years, SD = 6.34) participants. The mean length of storage time before SomaScan protein quantification was 3.84 years (SD = 1.32).

**TABLE 2:** Demographic information by amyloid status (amyloid negative or amyloid positive) for the clinically unimpaired, independent Stanford and Aging Memory Study cohort (SAMS CU cohort).

	Overall, N = 147^1^	Negative, N = 109^1^	Positive, N = 38^1^
Age, years	68.69 (5.79)	68.1 (5.5)	70.2 (6.3)
Gender			
Female	90 (61%)	66 (61%)	24 (63%)
Male	57 (39%)	43 (39%)	14 (37%)
Length of CSF storage time, years	3.83 (1.32)	3.88 (1.29)	3.69 (1.39)
Aß_42_/Aß_40_ ratio	0.09 (0.02)	0.100 (0.010)	0.054 (0.012)
MMSE	29.11 (0.90)	29.16 (0.87)	28.95 (0.96)
Unknown	5	5	0
p‐tau181	39.75 (21.05)	33 (9)	60 (31)
White matter hypointensity volume	2264.96 (2,182.09)	2171 (2,194)	2534 (2,155)
Unknown	4	3	1
Memory composite	0.09 (0.75)	0.17 (0.68)	−0.13 (0.88)
Unknown	6	6	0
Executive function composite	0.06 (0.73)	0.17 (0.67)	−0.21 (0.81)
Unknown	6	6	0
*APOE* ε4 allele count			
0	95 (77%)	78 (86%)	17 (52%)
1	26 (21%)	13 (14%)	13 (39%)
2	3 (2.4%)	0 (0%)	3 (9.1%)
Unknown	23	18	5
Total gray matter volume	601,534.04 (49,725.45)	602,657 (50,864)	598,317 (46,828)
Unknown	4	3	1
Hippocampal volume	7941.23 (769.82)	7995 (767)	7787 (766)
Unknown	4	3	1

^1^ Mean standard deviation (SD); *n* (%).

We used a module preservation analysis to determine whether the co‐expression network could be reproduced within our SAMS CU cohort. Fifteen modules—including nine AD‐relevant modules—were highly preserved, with Z_Summary_ values ranging from 12.0 to 31.0 (Figure S4). All remaining modules were weakly preserved, with Z_Summary_ values ranging from 4.0 to 9.4.

### 3.4 Modules M4‐phosphorylation, M7‐ubiquitination, and M18‐steroid dehydrogenase activity predict amyloid status and clinical phenotypes

**FIGURE 3:**
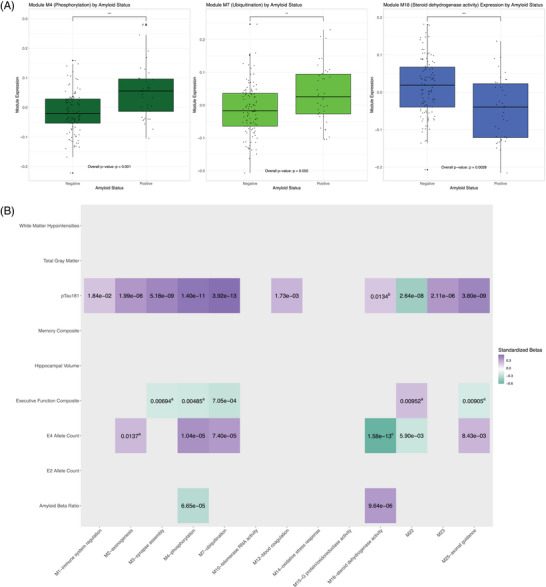
Module/phenotype relationships. (A) Modules by amyloid status. Box plots illustrating the results of Kruskall–Wallis tests for one‐way ANOVA used to calculate module eigenprotein relationships to amyloid status within the independent SAMS CU cohort. From left to right, modules M4‐phosphorylation, M7‐ubiquitination and M18‐steroid dehydrogenase activity are depicted. (B) Heatmap visualizing module relationships to cognition, AD pathology, genotype, and structural MRI outcomes within the independent SAMS CU cohort. Only module/phenotype relationships significant after multiple comparison correction are depicted. Heatmap colors range from purple to turquoise to red, reflecting the magnitude and direction of standardized beta values. The text within heat map cells are the unadjusted *p*‐values for each association. Module/trait relationships whose unadjusted *p*‐values change in significance (or are otherwise noteworthy) after controlling for amyloid and/or tau are marked with different superscripts: those that lose significance after controlling for amyloid are marked with "a”; those that gain significance after controlling for amyloid are marked with “b”; those that maintain significance after controlling for amyloid and tau are marked with “c.”

We used Kruskal–Wallis tests to assess module relationships to Aβ status (Figure 3A) and found M4‐phosphorylation (*p* = 0.0007) and M18‐steroid dehydrogenase activity (*p* = 0.0028) to be significantly associated with amyloid positivity after FDR correction. These modules remained associated with amyloid when CSF Aβ42/Aβ40 ratios were treated continuously and adjusted for age and sex, again after FDR correction (M4‐phosphorylation: *β* = −0.09, *p* = 0.001; M18‐steroid dehydrogenase activity: *β* = 0.10, *p* < 0.001) (Figure 3B). Module M7‐ubiquitination (*p* = 0.0066, FDR‐corrected: *p* = 0.0550) also had a significant association with amyloid status before—as well as a suggestive association after—multiple comparison correction.

To understand how modules might contribute to other phenotypes relevant to aging and AD risk, we focused on the associations between AD‐relevant modules and continuous CSF p‐tau181, composite cognitive scores, *APOE* genotype, and structural MRI measures (Figure 3B, Tables S1–S3). Ten of the 13 AD‐relevant were associated with p‐tau181 levels: M1‐immune system regulation, M2‐axonogenesis, M3‐synapse assembly, M4‐phosphorylation, M7‐ubiquitination, M12‐blood coagulation, M22, M23, and M25‐axonal guidance. Six of the AD‐relevant modules were associated with ε4 allele count: M2‐axonogenesis, M4‐phosphorylation, M7‐ubiquitination, M18‐steroid dehydrogenase activity, M22, and M25‐axonal guidance. Five modules were associated with executive function composite scores: M3‐synapse assembly, M4‐phosphorylation, M7‐ubiquitination, M22, and M25‐axonal guidance.

There was little difference in results between demographically adjusted (Figure 3B, Table S1) and amyloid‐adjusted models (Figure 3B, Table S2). After controlling for continuous Aβ42/Aβ40 values, M18‐steroid dehydrogenase activity was additionally associated with CSF p‐tau181 levels, whereas M2‐axonogenesis was no longer associated with ε4 allele count. Only M7‐ubiquitination was associated with executive function composite scores in these analyses.

Finally, the only significant relationship that persisted after controlling for p‐tau181 and Aβ42/Aβ40 values was that between ε4 allele count and module M18‐steroid dehydrogenase activity (Figure 3B, Table S3). A number of relationships were significant before multiple comparison correction. Specifically, M3‐synapse assembly and M7‐ubiquitination were associated with executive function; M4‐phosphorylation and M7‐ubiquitination were associated with ε4 allele count; M10‐telomerase RNA activity was associated with hippocampal volume; and M2‐axonogenesis and M3‐synapse assembly were associated with white matter hypointensity volume (Table S3).

Many of these patterns remained significant even among Aβ− participants alone (Figure S4). All but M1‐immune system regulation remained associated with p‐tau181 levels, and M18‐steroid dehydrogenase activity remained associated with ε4 allele count. Many more modules were associated with continuous amyloid ratios among Aβ− participants, including M1‐immune system regulation, M2‐axonogenesis, M3‐synapse assembly, M7‐ubiquitination, M22, M23, and M25‐axon guidance (Figure S5).

### 3.5 Genetic and cell‐type module enrichment

**FIGURE 4:**
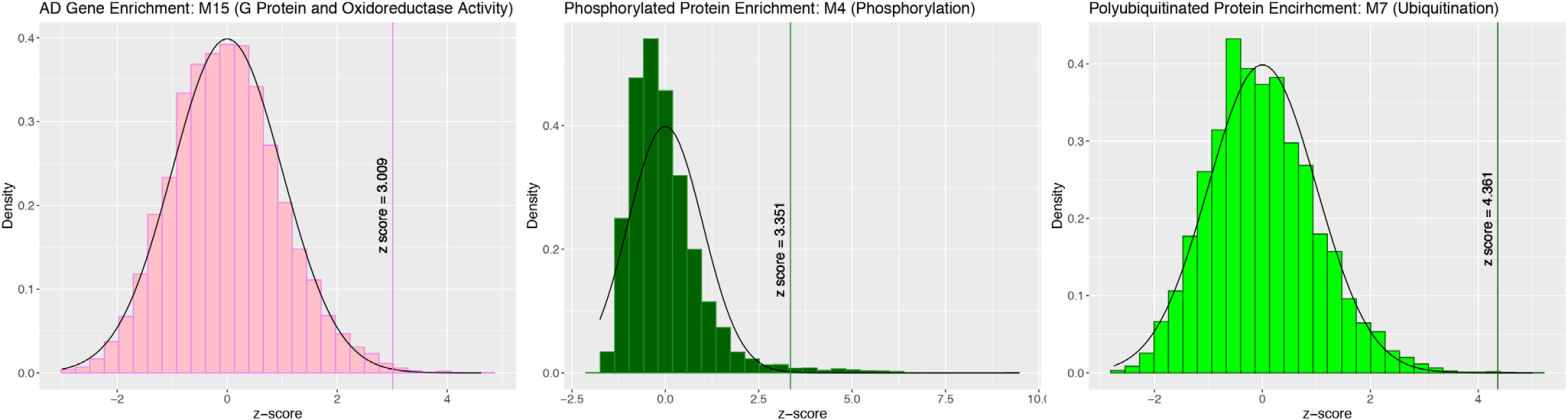
Module enrichment of AD genetic risk factors and post‐translationally modified proteins. Histograms representing the bootstrapped null distribution of either the average –log10 *p*‐values, number of ubiquitination sites, or number of protein phosphopeptides. These histograms were derived from randomly sampling a module‐sized collection of proteins 10,000 times. Histograms are overlaid with normal distribution curves, and vertical lines represent z scores capturing the distance between the average *p*‐value within a given module and its bootstrapped null distribution. Only vertical lines with z‐scores significant above the 90% confidence interval critical value (−1.645 or 1.645) are labeled. Significant enrichment results in module M15‐G protein and oxidoreductase activity of gene‐level associations with clinical AD dementia from the Bellenguez et al. (2022)^39^ GWAS, module M4‐phosphorylation of protein phosphopeptides derived from the Ping et al. (2020)^42^ mapping of the phosphoproteome, and module M7‐ubiquitination of polyubiquitinated proteins derived from the Abreha et al. (2018)^41^ mapping of the ubiquitylome.

Next, we explored whether any AD‐relevant modules were enriched for genetic variants associated with AD risk in GWASs. First, we examined whether they were enriched for proteins expressed by genetic regulators of clinical AD dementia (Kunkle et al.,^38^; Bellenguez et al.,^39^) and/or amyloid burden measured with PET (Raghavan et al.,^37^), as established by various GWAS summary statistics. We used *Z* scores to determine how the average log‐transformed *p*‐value within our module compared to a distribution of 10,000 module‐sized random samples. M13‐G protein and oxidoreductase activity (*Z* = 3.009) was significantly enriched for proteins associated with genetic regulators of clinical AD dementia (Figure 3). In addition, in an effort to validate M4‐phosphorylation and M7‐ubiquitination module functional annotations, we sought to ensure that these modules were enriched for such post‐translationally modified proteins. Using a mapping of the ubiquitylome by Abreha et al. (2018) and of the phosphoproteome by Ping et al. (2020), we confirmed that M4‐phosphorylation was significantly enriched for protein phosphopeptides (*Z* = 3.351), whereas M7‐ubiquitination was enriched with polyubiquitinated proteins (*Z* = 4.361) (Figure 4).

We also performed cell‐type enrichment analyses on our AD‐relevant modules, using the internet‐based application, WebCSEA. Eight of our 13 modules were enriched for specific cell types after Bonferroni correction: M2‐axonogenesis for macrophages, M3‐synapse assembly for neurons, M4‐phosphorylation for neurons, M7‐ubiquitination for excitatory neurons and stromal cells, M10‐telomerase RNA activity for enterocytes and red blood cells, M15‐G protein/oxidoreductase activity, M18‐steroid dehydrogenase activity for epithelial and red blood cells, and M25‐axon guidance for stromal cells (Figure S6).

### 3.6 Proteins within modules M4‐phosphorylation and M7‐ubiquitination accurately predict amyloid status in an independent CU cohort

Given the relationship between amyloid status and M4‐phosphorylation, M7‐ubiquitination, and M18‐steroid dehydrogenation activity, we sought to understand whether individual proteins within this module could accurately predict abnormal amyloid accumulation among a test set of 54 ADRC+ CU participants whose data were not used for network construction (Tables 3, 4, 5). For each of these modules, we performed multivariate LASSO regression on a model derived from module proteins, age, sex, and *APOE* ε4 allele count.

**TABLE 3:** Demographic information by amyloid status (amyloid negative or amyloid positive) for the clinically unimpaired, Stanford and Aging Memory Study (SAMS CU cohort) participants used to train LASSO and logistic regression models.

	Overall, N = 118^1^	Negative, N = 86^1^	Positive, N = 32^1^
Age, years	68.88 (6.02)	68.23 (5.64)	70.62 (6.71)
Gender			
Female	72 (61%)	53 (62%)	19 (59%)
Male	46 (39%)	33 (38%)	13 (41%)
Length of CSF storage time, years	3.81 (1.31)	3.89 (1.27)	3.60 (1.41)
*APOE* ε allele count			
0	72 (72%)	60 (82%)	12 (44%)
1	25 (25%)	13 (18%)	12 (44%)
2	3 (3.0%)	0 (0%)	3 (11%)
Unknown	18	13	5

^1^ Mean standard deviation (SD); *n* (%).

**TABLE 4:** Demographic information by amyloid status (amyloid negative or amyloid positive) for the clinically unimpaired, Stanford and Aging Memory Study (SAMS CU cohort) participants used to validate LASSO and logistic regression models.

	Overall, N = 29^1^	Negative, N = 23^1^	Positive, N = 6^1^
Age, years	67.90 (4.78)	67.83 (5.14)	68.17 (3.43)
Gender			
F	18 (62%)	13 (57%)	5 (83%)
M	11 (38%)	10 (43%)	1 (17%)
Length of CSF storage time, years	3.94 (1.36)	3.87 (1.40)	4.20 (1.28)
*APOE* ε4 allele count			
0	28 (95.8%)	23 (100%)	5 (83%)
1	1 (4.2%)	0 (0%)	1 (17%)
2	0 (0%)	0 (0%)	0 (0%)
Unknown	5	5	0

^1^ Mean standard deviation (SD); *n* (%).

**TABLE 5:** Demographic information by amyloid status (amyloid negative or amyloid positive) for the clinically unimpaired participants from the ADRC+ cohort used to test LASSO and logistic regression models.

	Overall, N = 54^1^	Negative, N = 29^1^	Positive, N = 25^1^
Age, years	68.44 (8.47)	67.76 (7.74)	69.24 (9.35)
Gender			
F	28 (52%)	15 (52%)	13 (52%)
M	26 (48%)	14 (48%)	12 (48%)
Sample origin			
ADRC participant	40 (74%)	17 (59%)	23 (92%)
Clinic non‐enrollee	14 (26%)	12 (41%)	2 (8.0%)
Length of CSF storage time, years	3.73 (2.16)	4.37 (2.21)	3.00 (1.88)
*APOE* ε4 allele count			
0	21 (52%)	12 (71%)	9 (39%)
1	12 (30%)	4 (24%)	8 (35%)
2	7 (18%)	1 (5.9%)	6 (26%)
Unknown	14	12	2

^1^ Mean standard deviation (SD); *n* (%).

**FIGURE 5:**
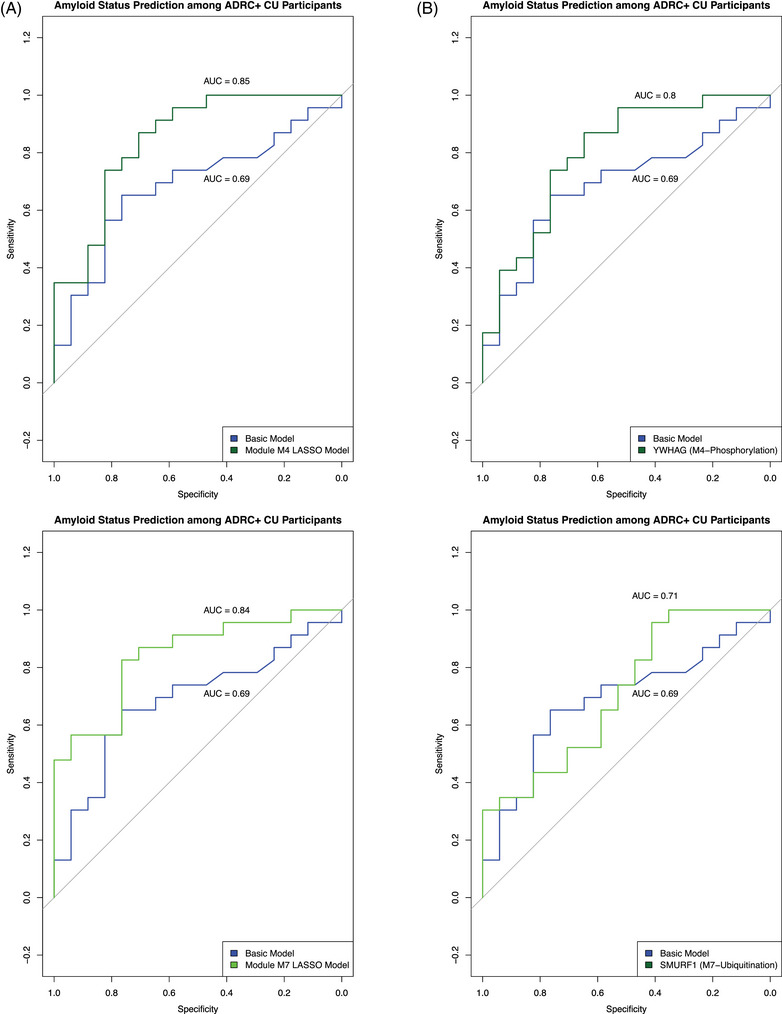
Module M3's prediction of amyloid status using LASSO regression with stability selection. (A) Receiver‐operating characteristic (ROC) curves depicting the classification performance (sensitivity vs specificity) of models predicting amyloid status among a test set of CU ADRC+ participants. In blue are results from a basic logistic regression model including *APOE* ε4 allele count, sex, and age (AUC = 0.71). In either dark green or green are results from a LASSO regression model derived from module M4‐phosphorylation or M7‐ubiquitination proteins, respectively, along with the previously mentioned demographic and genotype factors. (C) ROC curves similar to those in (A), except in dark green or green are results from a logistic regression model including only stable model features, along with *APOE* ε4 allele count: YWHAG for M4‐phosphorylation and SMURF1 for M7‐ubiquitination.

Both M4‐phosphorylation (AUC = 0.85, 95% confidence interval [CI] = 0.72–0.97, seven‐parameter solution) and M7‐ubiquitination (AUC = 0.84, 95% CI = 0.72–0.96, six‐parameter solution) predicted amyloid status with high accuracy among the test set of ADRC+ CU participants. (Figure 5A). In contrast, a logistic regression containing only *APOE* ε4 allele count, age, and sex weakly predicted amyloid status among ADRC+ CU participants (AUC = 0.69, 95% CI = 0.52–0.86). Module M18‐steroid dehydrogenase activity did not significantly predict amyloid status (AUC = 0.56, 95% CI = 0.38–0.75, 13‐parameter solution) (Figure S7A).

LASSO regression is a useful method of feature selection. It minimizes the loss function by reducing the absolute value of the sum of the model's coefficients, shrinking the coefficients of weak and redundant parameters to 0. We used a stability selection procedure to determine which variables were most likely to be selected across many different iterations of LASSO regression, using a selection probability greater than 65% as our cutoff. The stably selected variables included YWHAG for module M4‐phosphorylation and SMURF1 and *APOE* ε4 allele count for module M7‐ubiquitination (Figure S7B,C). We used the stably selected proteins as predictors in separate logistic regression models that additionally controlled for *APOE* ε4 allele count. These models predicted amyloid status with moderate to weak accuracy (YWHAG [M4‐phosphorylation]—AUC = 0.80, 95% CI = 0.65–0.94; SMURF1 [M7‐ubiquitination]—AUC = 0.71, 95% CI = 0.55–0.88) (Figure 5B).

## 4 DISCUSSION

In this study, we used CSF proteins to construct a co‐expression network among a cohort of individuals along the clinical AD continuum and replicated this network in an independent cohort of CU older adults. We further examined the relationship between protein clusters—or modules—within this network and phenotypes relevant to aging and AD, such as CSF measures of amyloid and tau burden, cognition, structural neuroimaging outcomes, and *APOE* genotype. This approach allowed us to identify modules relevant to AD disease biology and evaluate their early functional and physiological consequences among CU individuals.

The modules we observed resembled those described previously in larger‐scale proteomic studies. These include modules devoted to axonal development, blood coagulation, RNA activity, synapse assembly, G protein and oxidoreductase activity, myelination, and protein kinase activity.^22^, ^23^, ^26^, ^28^, ^49^ Modules M3‐synapse assembly, M4‐phosphorylation, M10‐telomerase RNA activity, and M23 were associated with clinical disease stage after multiple comparison correction.

In addition, modules M4‐phosphorylation, M7‐ubiquitination, and M18‐steroid dehydrogenase activity were associated with abnormal Aβ aggregation within the SAMS cohort. Although not associated with clinical diagnosis, these modules arguably reflect early changes in the AD cascade and are relevant to understanding disease biology. These modules were also associated with p‐tau181 levels, particularly after adjusting for amyloid pathology. Modules M4‐phosphorylation and M7‐ubiquitination showed amyloid‐independent effects with *APOE* ε4 genotype, whereas M18‐steroid dehydrogenase activity had a tau and amyloid‐independent effect on genotype. Module M4‐ubiquitination was further associated with executive function.

We performed enrichment analyses and found that only module M15‐G protein and oxidoreductase activity were enriched for proteins associated with genetic regulators of clinical AD dementia.^39^ We additionally confirmed that modules M4‐phosphorylation and M7‐ubiquitination were enriched with such post‐translationally modified proteins (i.e., protein phosphopeptides and polyubiquitinated proteins, respectively). Furthermore, we performed cell‐type enrichment analyses on our AD‐relevant modules and found them to be enriched for neuronal, stromal, macrophage, epithelial, and red blood cell types.

Using LASSO regression analyses, we observed that modules M4‐phosphorylation and M7‐ubiquitination accurately predicted amyloid status among 54 CU ADRC+ participants who were not included in the network construction process, with AUCs of 0.85 and 0.84, respectively. A logistic regression model that included 14‐3‐3 protein gamma (YWHAG)—a stably selected protein from the M4‐phosphorylation module—outperformed one that included *APOE* ε4 genotype, age, and sex, alone (AUC of 0.80 vs an AUC of 0.69).

Our findings underscore the importance of protein post‐translational modification in abnormal amyloid accumulation. The role of post‐translational modifications—such as phosphorylation and ubiquitination—in AD have been described in detail.^41^, ^50^, ^51^ As mentioned, neurofibrillary tangles in AD comprise the hyperphosphorylated tau protein; in addition, Aβ production can be regulated by the phosphorylation of the amyloid precursor protein [APP].^50^ A number of 14‐3‐3 proteins—including YWHAG, YWHAE, and YWHAB—were members of the M4‐phosphorylation module. These phospho‐binding proteins regulate a wide range of functions within the brain, including protein kinase activity, apoptosis, cell trafficking, and neuronal plasticity.^52^ In addition, there is evidence that these proteins interact with tau and can promote its phosphorylation.^53^ M4‐phosphorylation also contained Ca^2+^/calmodulin‐dependent protein kinases (CAMK2B and CAMK2D), which had relatively high selection probabilities for an M4‐wide LASSO regression model. These calcium‐signaling molecules have been linked to both phosphorylation of the tau protein and APP.^54^

M7 was enriched for ubiquitination, a post‐translational modification mediated by a sequential cascade of enzymes that transfer ubiquitin, a 76 amino acid protein, to lysine residues on target proteins. Ubiquitin can be assembled into polymeric chains via ubiquitination of one of its seven lysine (K) residues: K6, K11, K27, K29, K33, K48, and K63.^55^ M7 was enriched specifically for K63‐linked ubiquitination, which is involved in non‐proteasomal functions, such as protein kinase activation, DNA repair, and autophagy.^56^ Autophagy is a degradative process mediated by the lysosome and critical to the cellular response to stress, such as nutrient starvation, hypoxia, oxidative stress, and DNA damage.^57^, ^58^ It degrades misfolded proteins—particularly long‐lived, insoluble, protein aggregates,^59^—as well as damaged organelles.^57^ M7‐ubiquitination contained a number of regulators of autophagy machinery, such as MAP1LC3A, GABARAP, GABARAPL1, and GABARAPL2.^60^

Autophagy induced by nutrient starvation is meant to promote cell survival, by providing cells with internal nutrient supplies and clearing protein aggregates.^58^, ^61^ However, there is evidence to suggest that autophagy is dysregulated in AD.^62^, ^63^ In a 5fXAD mouse model of AD, fasting led to an increase in macroautophagy activity, but did not result in subsequent degradation of intracellular Aβ accumulation that stemmed from increased extracellular uptake.^64^ Although we cannot establish the direction of causality between autophagy and AD pathology, it is plausible that increased autophagy is detectable in the CU stages preceding clinical impairment.

In conclusion, by examining module changes in the absence of clinical impairment, our study enabled us to elucidate the critical importance of phosphorylation and ubiquitination for preclinical changes in cognition and pathology. The focus on abnormal amyloid aggregation is particularly relevant because clinical trials are increasingly targeting this earliest stage of disease for therapeutic intervention. By leveraging a large sample of CU participants and cutting‐edge protein quantification technology, we were able to identify biological mechanisms associated with amyloid positivity. The SOMAScan platform is the largest protein panel available for clinical screening of CSF samples, and its aptamer‐based technology enabled high‐throughput protein quantification. Although some studies have observed the potential for aptamer off‐target cross‐reactivity with homologous proteins, they have also noted that in this context, roughly half of the time aptamers are binding to alternative forms of the same protein.^65^, ^66^ In addition, our reliance on network analytical approaches provided an additional safeguard against such concerns.

Our study has several limitations. This work is cross‐sectional, and longitudinal studies are needed to understand the time course of these proteomic signatures and the ability of these modules to predict future progression from CU to clinical impairment (MCI and AD dementia). Furthermore, we were unable to functionally annotate modules with important relationships to AD pathology, such as M22 and M23, possibly due to their extreme module sizes. Finally, our cohort is predominantly non‐Hispanic White and highly educated, thereby limiting the generalizability of our findings. Despite these limitations, our work relating CSF protein modules and phenotypes relevant to aging and AD dementia is important given the need to discover mechanisms driving initial disease processes in the absence of clinical impairment. Overall, our study highlights the important, multi‐faceted involvement of ubiquitination in the AD cascade, particularly at its initial stages.

## AUTHOR CONTRIBUTIONS

O.A. and E.C.M. designed and conceptualized the study. A.D.W., S.J.S., K.I.A., K.L.P., V.W.H., T.W.‐C., and E.C.M. provided the data. O.A. led and performed all data analysis. J.R. and P.M.‐L. contributed to data analysis. M.E.B. and Z.H. aided in statistical analyses. E.N.W. led Alzheimer's disease CSF biomarker collection and quantification. A.N.T., D.C., A.R., and J.P. assisted in data collection. O.A. and E.C.M. drafted and substantively revised the manuscript. All authors read and provided comments on an earlier draft and approved the final manuscript.

## CONFLICT OF INTEREST STATEMENT

E.C.M. is a paid consultant for Roche, Genentech, and Eli Lilly. T.W.‐C. is a co‐founder and scientific advisor of Alkahest Inc and Qinotto Inc. T.W.‐C. and J.R. are co‐founders and scientific advisors of Teal Omics Inc. All other authors have no disclosures relevant to this manuscript. Author disclosures are available in the Supporting Information.

## CONSENT STATEMENT

All ADRC+ and SAMS participants provided written informed consent in compliance with local institutional review boards.

## Supporting information

Supporting Information

Supporting Information

Supporting Information

Supporting Information

Supporting Information

Supporting Information

Supporting Information

Supporting Information

Supporting Information

Supporting Information

Supporting Information

Supporting Information

## References

[1] Alzheimer's Association . 2020 Alzheimer's Disease facts and figures. 2020.

[2] Holtzman DM , Morris JC , Goate AM . Alzheimer's disease: the challenge of the second century. Sci Transl Med. 2011;3. 77sr1. 10.1126/scitranslmed.3002369 PMC3130546 21471435

[3] Chiti F , Dobson CM . Protein misfolding, functional amyloid, and human disease. Annu Rev Biochem. 2006;75:333‐366. 16756495 10.1146/annurev.biochem.75.101304.123901

[4] Morozova OA , March ZM , Robinson AS , Colby DW . Conformational features of tau fibrils from Alzheimer's disease brain are faithfully propagated by unmodified recombinant protein. Biochemistry. 2013;52:6960‐6967. 24033133 10.1021/bi400866w PMC4142060

[5] Myeku N , Duff KE . Targeting the 26S proteasome to protect against proteotoxic diseases. Trends Mol Med. 2018;24:18‐29. 29233753 10.1016/j.molmed.2017.11.006 PMC5905406

[6] Sperling RA , Aisen PS , Beckett LA , et al. Toward defining the preclinical stages of Alzheimer's disease: recommendations from the National Institute on Aging‐Alzheimer's Association workgroups on diagnostic guidelines for Alzheimer's disease. Alzheimers Dement. 2011;7:280‐292. 21514248 10.1016/j.jalz.2011.03.003 PMC3220946

[7] Jack CR Jr , Bennett DA , Blennow K , et al. NIA‐AA Research Framework: toward a biological definition of Alzheimer's disease. Alzheimers Dement. 2018;14:535‐562. 29653606 10.1016/j.jalz.2018.02.018 PMC5958625

[8] Doré V , Villemagne VL , Bourgeat P , et al. Cross‐sectional and longitudinal analysis of the relationship between Aβ deposition, cortical thickness, and memory in cognitively unimpaired individuals and in Alzheimer disease. JAMA Neurol. 2013;70:903‐911. 23712469 10.1001/jamaneurol.2013.1062

[9] Wang L , Benzinger TL , Hassenstab J , et al. Spatially distinct atrophy is linked to β‐amyloid and tau in preclinical Alzheimer disease. Neurology. 2015;84:1254‐1260. 25716355 10.1212/WNL.0000000000001401 PMC4366088

[10] Sperling RA , Mormino EC , Schultz AP , et al. The impact of amyloid‐beta and tau on prospective cognitive decline in older individuals. Ann Neurol. 2019;85:181‐193. 30549303 10.1002/ana.25395 PMC6402593

[11] Mormino EC , Papp KV , Rentz DM , et al. Early and late change on the preclinical Alzheimer's cognitive composite in clinically normal older individuals with elevated amyloid β. Alzheimers Dement. 2017;13:1004‐1012. 28253478 10.1016/j.jalz.2017.01.018 PMC5573651

[12] Insel PS , Weiner M , Mackin RS , et al. Determining clinically meaningful decline in preclinical Alzheimer disease. Neurology. 2019;93:e322‐e333. 31289148 10.1212/WNL.0000000000007831 PMC6669933

[13] Jack CR Jr , Knopman DS , Jagust WJ , et al. Hypothetical model of dynamic biomarkers of the Alzheimer's pathological cascade. Lancet Neurol. 2010;9:119‐128. 20083042 10.1016/S1474-4422(09)70299-6 PMC2819840

[14] Palmqvist S , Mattsson N , Hansson O . Alzheimer's Disease Neuroimaging Initiative. Cerebrospinal fluid analysis detects cerebral amyloid‐β accumulation earlier than positron emission tomography. Brain. 2016;139:1226‐1236. 26936941 10.1093/brain/aww015 PMC4806222

[15] Sperling R , Mormino E , Johnson K . The evolution of preclinical Alzheimer's disease: implications for prevention trials. Neuron. 2014;84:608‐622. 25442939 10.1016/j.neuron.2014.10.038 PMC4285623

[16] Bai B , Vanderwall D , Li Y , et al>. Proteomic landscape of Alzheimer's Disease: novel insights into pathogenesis and biomarker discovery. Mol Neurodegener. 2021;16:55. 34384464 10.1186/s13024-021-00474-z PMC8359598

[17] Palstrøm NB , Matthiesen R , Rasmussen LM , Beck HC . Recent developments in clinical plasma proteomics‐applied to cardiovascular research. Biomedicines. 2022;10(1):162. doi:10.3390/biomedicines10010162 35052841 PMC8773619

[18] Higginbotham L , Dammer EB , Duong DM , et al. Network analysis of a membrane‐enriched brain proteome across stages of Alzheimer's disease. Proteomes. 2019;7(3):30. doi:10.3390/proteomes7030030 31461916 PMC6789842

[19] Dai J , Johnson ECB , Dammer EB , et al. Effects of APOE genotype on brain proteomic network and cell type changes in Alzheimer's disease. Front Mol Neurosci. 2018;11:454. 30618606 10.3389/fnmol.2018.00454 PMC6305300

[20] Dammer EB , Ping L , Duong DM , et al. Multi‐platform proteomic analysis of Alzheimer's disease cerebrospinal fluid and plasma reveals network biomarkers associated with proteostasis and the matrisome. Alzheimers Res Ther. 2022;14:1‐32. 36384809 10.1186/s13195-022-01113-5 PMC9670630

[21] Johnson ECB , Dammer EB , Duong DM , et al. Deep proteomic network analysis of Alzheimer's disease brain reveals alterations in RNA binding proteins and RNA splicing associated with disease. Mol Neurodegener. 2018;13:52. 30286791 10.1186/s13024-018-0282-4 PMC6172707

[22] Johnson ECB , Dammer EB , Duong DM , et al. Large‐scale proteomic analysis of Alzheimer's disease brain and cerebrospinal fluid reveals early changes in energy metabolism associated with microglia and astrocyte activation. Nat Med. 2020;26:769‐780. 32284590 10.1038/s41591-020-0815-6 PMC7405761

[23] Johnson ECB , Carter EK , Dammer EB , et al. Large‐scale deep multi‐layer analysis of Alzheimer's disease brain reveals strong proteomic disease‐related changes not observed at the RNA level. Nat Neurosci. 2022;25(2):213‐225. 35115731 10.1038/s41593-021-00999-y PMC8825285

[24] Seyfried NT , Dammer EB , Swarup V , et al. A multi‐network approach identifies protein‐specific co‐expression in asymptomatic and symptomatic Alzheimer's disease. Cell Systems. 2017;4(1):60‐72. e4. 27989508 10.1016/j.cels.2016.11.006 PMC5269514

[25] Swarup V , Chang TS , Duong DM , et al>. Identification of conserved proteomic networks in neurodegenerative dementia. Cell Rep. 2020;31:107807. 32579933 10.1016/j.celrep.2020.107807 PMC8221021

[26] Higginbotham L , Ping L , Damme EB , et al. Integrated proteomics reveals brain‐based cerebrospinal fluid biomarkers in asymptomatic and symptomatic Alzheimer's disease. Sci Adv. 2020;6(43):eaaz9360. 33087358 10.1126/sciadv.aaz9360 PMC7577712

[27] Anderson ND . State of the science on mild cognitive impairment (MCI). CNS Spectr. 2019;24:78‐87. 30651152 10.1017/S1092852918001347

[28] Bai B , Wang X , Li Y , et al. Deep multilayer brain proteomics identifies molecular networks in Alzheimer's disease progression. Neuron. 2020;105:975‐991. e7. 31926610 10.1016/j.neuron.2019.12.015 PMC7318843

[29] Wilson EN , Young CB , Ramos Benitez J , et al. Performance of a fully‐automated Lumipulse plasma phospho‐tau181 assay for Alzheimer's disease. Alzheimers Res Ther. 2022;14:1‐12. 36371232 10.1186/s13195-022-01116-2 PMC9652927

[30] Trelle AN , Carr VA , Guerin SA , et al. Hippocampal and cortical mechanisms at retrieval explain variability in episodic remembering in older adults. Elife. 2020;9. doi:10.7554/eLife.55335 PMC7259949 32469308

[31] Trelle AN , Carr VA , Wilson EN , et al. Association of CSF biomarkers with hippocampal‐dependent memory in preclinical Alzheimer disease. Neurology. 2021;96:e1470‐e1481. 33408146 10.1212/WNL.0000000000011477 PMC8055319

[32] Shuken SR , Rutledge J , Iram T , et al. Limited proteolysis – mass spectrometry reveals aging‐associated changes in cerebrospinal fluid protein abundances and structures. Nature Aging. 2022;2:379‐388. 36741774 10.1038/s43587-022-00196-x PMC9893943

[33] Gold L , Ayers D , Bertino J , et al. Aptamer‐based multiplexed proteomic technology for biomarker discovery. PLoS One. 2010;5:e15004. 21165148 10.1371/journal.pone.0015004 PMC3000457

[34] Langfelder P , Horvath S . WGCNA: an R package for weighted correlation network analysis. BMC Bioinformatics. 2008;9:559. 19114008 10.1186/1471-2105-9-559 PMC2631488

[35] Poplin R , Ruano‐Rubio V , DePristo MA , et al. Scaling accurate genetic variant discovery to tens of thousands of samples. bioRxiv. 2018. doi:10.1101/201178

[36] Langfelder P , Luo R , Oldham MC , Horvath S . Is my network module preserved and reproducible? PLoS Comput Biol. 2011;7:e1001057. 21283776 10.1371/journal.pcbi.1001057 PMC3024255

[37] Raghavan NS , Dumitrescu L , Mormino E , et al. Association between common variants in RBFOX1, an RNA‐binding protein, and brain amyloidosis in early and preclinical Alzheimer disease. JAMA Neurol. 2020;77:1288‐1298. 32568366 10.1001/jamaneurol.2020.1760 PMC7309575

[38] Kunkle BW , Grenier‐Boley B , Sims R , et al. Genetic meta‐analysis of diagnosed Alzheimer's disease identifies new risk loci and implicates Aβ, tau, immunity and lipid processing. Nat Genet. 2019;51:414‐430. 30820047 10.1038/s41588-019-0358-2 PMC6463297

[39] Bellenguez C , Küçükali F , Jansen IE , et al. New insights into the genetic etiology of Alzheimer's disease and related dementias. Nat Genet. 2022;54:412‐436. 35379992 10.1038/s41588-022-01024-z PMC9005347

[40] Watanabe K , Taskesen E , van Bochoven A , Posthuma D . Functional mapping and annotation of genetic associations with FUMA. Nat Commun. 2017;8:1826. 29184056 10.1038/s41467-017-01261-5 PMC5705698

[41] Abreha MH , Dammer EB , Ping L , et al. Quantitative analysis of the brain ubiquitylome in Alzheimer's disease. Proteomics. 2018;18:e1800108. 30230243 10.1002/pmic.201800108 PMC6283072

[42] Ping L , Kundinger SR , Duong DM , et al. Global quantitative analysis of the human brain proteome and phosphoproteome in Alzheimer's disease. Sci Data. 2020;7:315. 32985496 10.1038/s41597-020-00650-8 PMC7522715

[43] Meinshausen N , Bühlmann P . Stability selection. J R Stat Soc Series B Stat Methodol. 2010;72:417‐473.

[44] Hofner B , Boccuto L , Göker M . Controlling false discoveries in high‐dimensional situations: boosting with stability selection. BMC Bioinformatics. 2015;16:144. 25943565 10.1186/s12859-015-0575-3 PMC4464883

[45] Mahoney ER , Dumitrescu L , Moore AM , et al. Brain expression of the vascular endothelial growth factor gene family in cognitive aging and alzheimer's disease. Mol Psychiatry. 2019;26:888‐896. 31332262 10.1038/s41380-019-0458-5 PMC6980445

[46] Whelan CD , Mattsson N , Nagle MW , et al. Multiplex proteomics identifies novel CSF and plasma biomarkers of early Alzheimer's disease. Acta Neuropathol Commun. 2019;7:169. 31694701 10.1186/s40478-019-0795-2 PMC6836495

[47] Teitsdottir UD , Jonsdottir MK , Lund SH , Darreh‐Shori T , Snaedal J , Petersen PH . Association of glial and neuronal degeneration markers with Alzheimer's disease cerebrospinal fluid profile and cognitive functions. Alzheimers Res Ther. 2020;12:1‐14. 10.1186/s13195-020-00657-8 PMC7404927 32753068

[48] Zhou M , Haque RU , Dammer EB , et al. Targeted mass spectrometry to quantify brain‐derived cerebrospinal fluid biomarkers in Alzheimer's disease. Clin Proteomics. 2020;17:19. 32514259 10.1186/s12014-020-09285-8 PMC7257173

[49] Walker KA , Chen J , Zhang J , et al. Large‐scale plasma proteomic analysis identifies proteins and pathways associated with dementia risk. Nat Aging. 2021;1:473‐489. 37118015 10.1038/s43587-021-00064-0 PMC10154040

[50] Oliveira J , Costa M , de Almeida MSC , da CE , Silva OAB , Henriques AG . Protein phosphorylation is a key mechanism in Alzheimer's disease. J Alzheimers Dis. 2017;58:953‐978. 28527217 10.3233/JAD-170176

[51] Ramesh M , Gopinath P , Govindaraju T . Role of post‐translational modifications in Alzheimer's disease. Chembiochem. 2020;21:1052‐1079. 31863723 10.1002/cbic.201900573

[52] Pair FS , Yacoubian TA . 14‐3‐3 Proteins: novel pharmacological targets in neurodegenerative diseases. Trends Pharmacol Sci. 2021;42:226‐238. 33518287 10.1016/j.tips.2021.01.001 PMC8011313

[53] Gu Q , Cuevas E , Raymick J , Kanungo J , Sarkar S . Downregulation of 14‐3‐3 proteins in Alzheimer's disease. Mol Neurobiol. 2020;57:32‐40. 31487003 10.1007/s12035-019-01754-y

[54] Ghosh A , Giese KP . Calcium/calmodulin‐dependent kinase II and Alzheimer's disease. Mol Brain. 2015;8:78. 26603284 10.1186/s13041-015-0166-2 PMC4657223

[55] Komander D . The emerging complexity of protein ubiquitination. Biochem Soc Trans. 2009;37:937‐953. 19754430 10.1042/BST0370937

[56] Puangmalai N , Sengupta U , Bhatt N , et al. Lysine 63‐linked ubiquitination of tau oligomers contributes to the pathogenesis of Alzheimer's disease. J Biol Chem. 2022;298:101766. 35202653 10.1016/j.jbc.2022.101766 PMC8942844

[57] Glick D , Barth S , Macleod KF . Autophagy: cellular and molecular mechanisms. J Pathol. 2010;221:3‐12. 20225336 10.1002/path.2697 PMC2990190

[58] Peker N , Gozuacik D . Autophagy as a cellular stress response mechanism in the nervous system. J Mol Biol. 2020;432:2560‐2588. 31962122 10.1016/j.jmb.2020.01.017

[59] Kocaturk NM , Gozuacik D . Crosstalk between mammalian autophagy and the ubiquitin‐proteasome system. Front Cell Dev Biol. 2018;6:128. 30333975 10.3389/fcell.2018.00128 PMC6175981

[60] Feng Y , He D , Yao Z , Klionsky DJ . The machinery of macroautophagy. Cell Res. 2014;24:24‐41. 24366339 10.1038/cr.2013.168 PMC3879710

[61] Shang L , Chen S , Du F , Li S , Zhao L , Wang X . Nutrient starvation elicits an acute autophagic response mediated by Ulk1 dephosphorylation and its subsequent dissociation from AMPK. Proc Natl Acad Sci U S A. 2011;108:4788‐4793. 21383122 10.1073/pnas.1100844108 PMC3064373

[62] Bagherniya M , Butler AE , Barreto GE , Sahebkar A . The effect of fasting or calorie restriction on autophagy induction: a review of the literature. Ageing Res Rev. 2018;47:183‐197. 30172870 10.1016/j.arr.2018.08.004

[63] Chatterjee S , Mudher A . Alzheimer's disease and type 2 diabetes: a critical assessment of the shared pathological traits. Front Neurosci. 2018;12:383. 29950970 10.3389/fnins.2018.00383 PMC6008657

[64] Chen X , Kondo K , Motoki K , Homma H , Okazawa H . Fasting activates macroautophagy in neurons of Alzheimer's disease mouse model but is insufficient to degrade amyloid‐beta. Sci Rep. 2015;5:12115. 26169250 10.1038/srep12115 PMC4648430

[65] Sun BB , Maranville JC , Peters JE , et al. Genomic atlas of the human plasma proteome. Nature. 2018;558:73‐79. 29875488 10.1038/s41586-018-0175-2 PMC6697541

[66] Williams SA , Kivimaki M , Langenberg C , et al. Plasma protein patterns as comprehensive indicators of health. Nat Med. 2019;25:1851‐1857. 31792462 10.1038/s41591-019-0665-2 PMC6922049

