# Supplementary material for: Post‐translational modifications linked to preclinical Alzheimer's disease–related pathological and cognitive changes
Source: Alzheimers Dement. 2023 Dec 25;20(3):1851–67. doi: 10.1002/alz.13576 (PMC10984434; doi:10.1002/alz.13576)
Supplement: Supplementary file 1 — Supporting Information [file ALZ-20-1851-s007.docx]

# **Supplementary Methods**

*Amyloid Status Determination*

Amyloid status was determined using CSF data quantified by either the (1) Lumipulse G system (Fujirebio US, Inc., Malvern, PA) ratios of Aβ_42_ to Aβ_40_ (*n* = 147 within the SAMS CU cohort and *n* = 89 within the ADRC+) or (2) the Quanterix Neurology 3-plex A assay (Quanterix ®, MA, USA) measures of Aβ_42_ (*n* =22 within the ADRC+ cohort).

There were three separate batches of Lumipulse data (one for the independent SAMS CU cohort, and two from the ADRC+ cohort). All batches included additional subjects not included in primary SomaScan data analyses. A previously-published Aβ_42_/Aβ_40_ ratio cutoff of 0.0752 was used to distinguish amyloid positive and amyloid negative SAMS participants [[1]](https://paperpile.com/c/FoTD2H/tiI9b). The two Lumipulse batches within the ADRC+ cohort correspond to 123 individuals enrolled within the Stanford Alzheimer’s Disease Research Center and to 66 participants recruited directly from an affiliated memory disorder clinic. Both batches included participants with non-AD diagnoses, including Parkinson’s disease (PD), Lewy body dementia, and MCI in PD; these participants were not included in SomaScan protein network analyses and used solely to determine amyloid status thresholds. To establish study-level cutoffs distinguishing amyloid positivity from negativity within these two batches, we performed Gaussian mixture modeling implemented by the R package mclust (version 5.4.7) separately for these two batches. Gaussian mixture models were estimated using a two-cluster solution with equal variance, and amyloid status cut-offs were determined by finding the Aβ_42_/Aβ_40_ ratio value corresponding to the 50% amyloid positive probability cluster. For the batch of 123 ADRC participants, we arrived at an Aβ_42_/Aβ_40_ ratio cutoff of 0.099 (**Fig. S1**), and for the batch of 66 memory clinic non-enrollees, we arrived at one of 0.088 (**Fig. S1**).

Twenty-two ADRC+ participants did not have Lumipulse-quantified CSF data available, but did have AD biomarkers quantified by the Quanterix Neurology 3-plex A assay (Quanterix ®, MA, USA). This data was used only to further determine amyloid status and was not used in any subsequent analyses of continuous amyloid. We again performed Gaussian mixture modeling using the same method as above, this time on 207 ADRC+ participants who had CSF Quanterix measurements. We arrived at an Aβ_42_/Aβ_40_ ratio cutoff of 0.048 (**Fig. S1**).

**References**

[1. Trelle AN, Carr VA, Wilson EN, Swarovski MS, Hunt MP, Toueg TN, et al. Association of CSF Biomarkers With Hippocampal-Dependent Memory in Preclinical Alzheimer Disease. Neurology. 2021;96:e1470–81.](http://paperpile.com/b/FoTD2H/tiI9b)


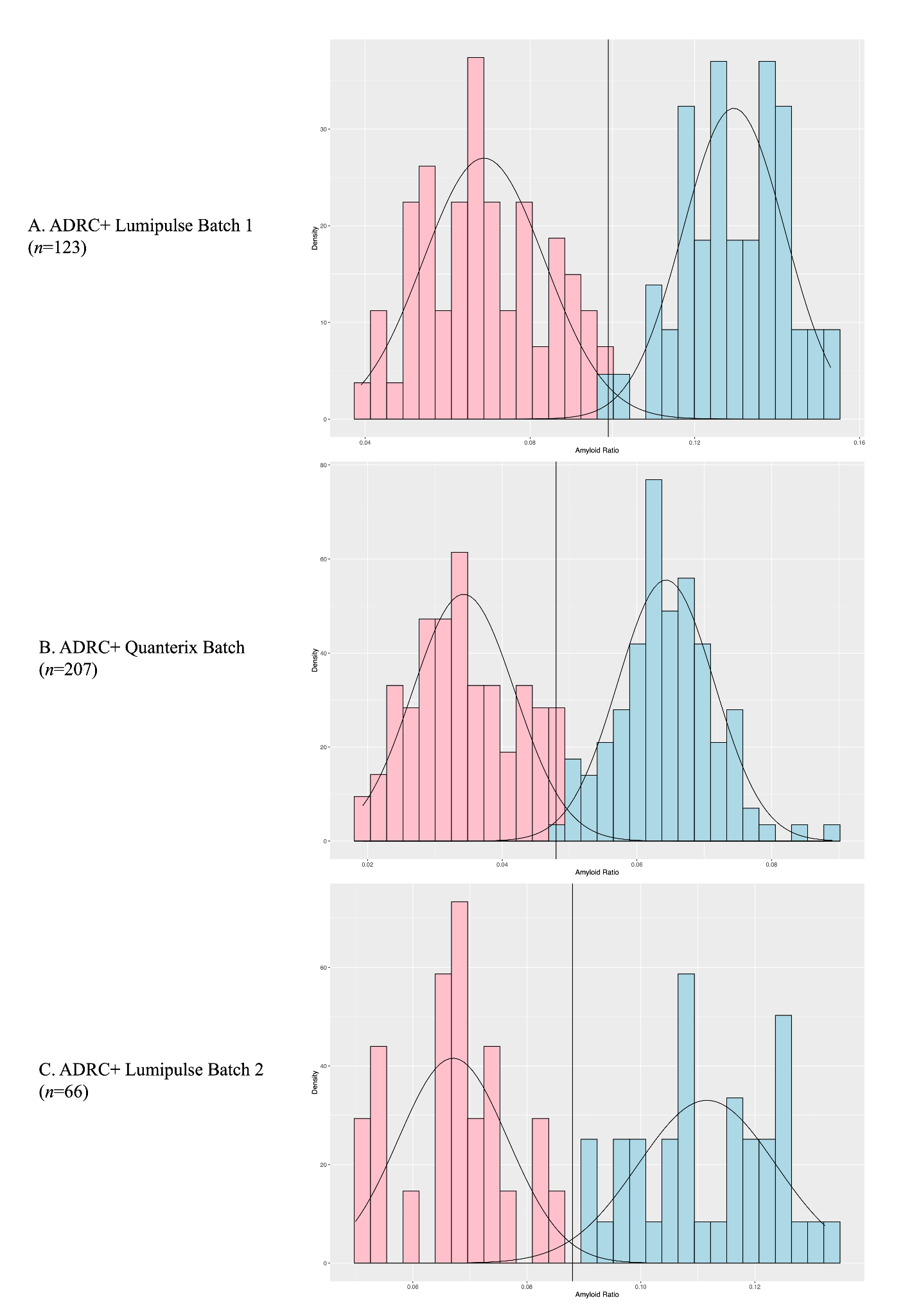


**Figure S1.** Distributions of amyloid ratio (Aβ_42_/Aβ_40_) values, superimposed with probability density functions for the estimated Gaussian distributions for a two-cluster model solution. The amyloid negative (Aβ-) distribution is in light blue, and the amyloid positive (Aβ+) distribution is in pink. Vertical lines represent the cutoff chosen that corresponds to the 50% amyloid positive probability cluster. (A) Results from the first ADRC+ batch quantified by Lumipulse (*n* = 123) (B) Results from the ADRC+ batch quantified by Quanterix (*n* = 207) (C) Results from the second ADRC+ batch quantified by Lumipulse (*n* = 66).
