# Supplementary material for: Post‐translational modifications linked to preclinical Alzheimer's disease–related pathological and cognitive changes
Source: Alzheimers Dement. 2023 Dec 25;20(3):1851–67. doi: 10.1002/alz.13576 (PMC10984434; doi:10.1002/alz.13576)
Supplement: Supplementary file 2 — Supporting Information [file ALZ-20-1851-s003.pdf]

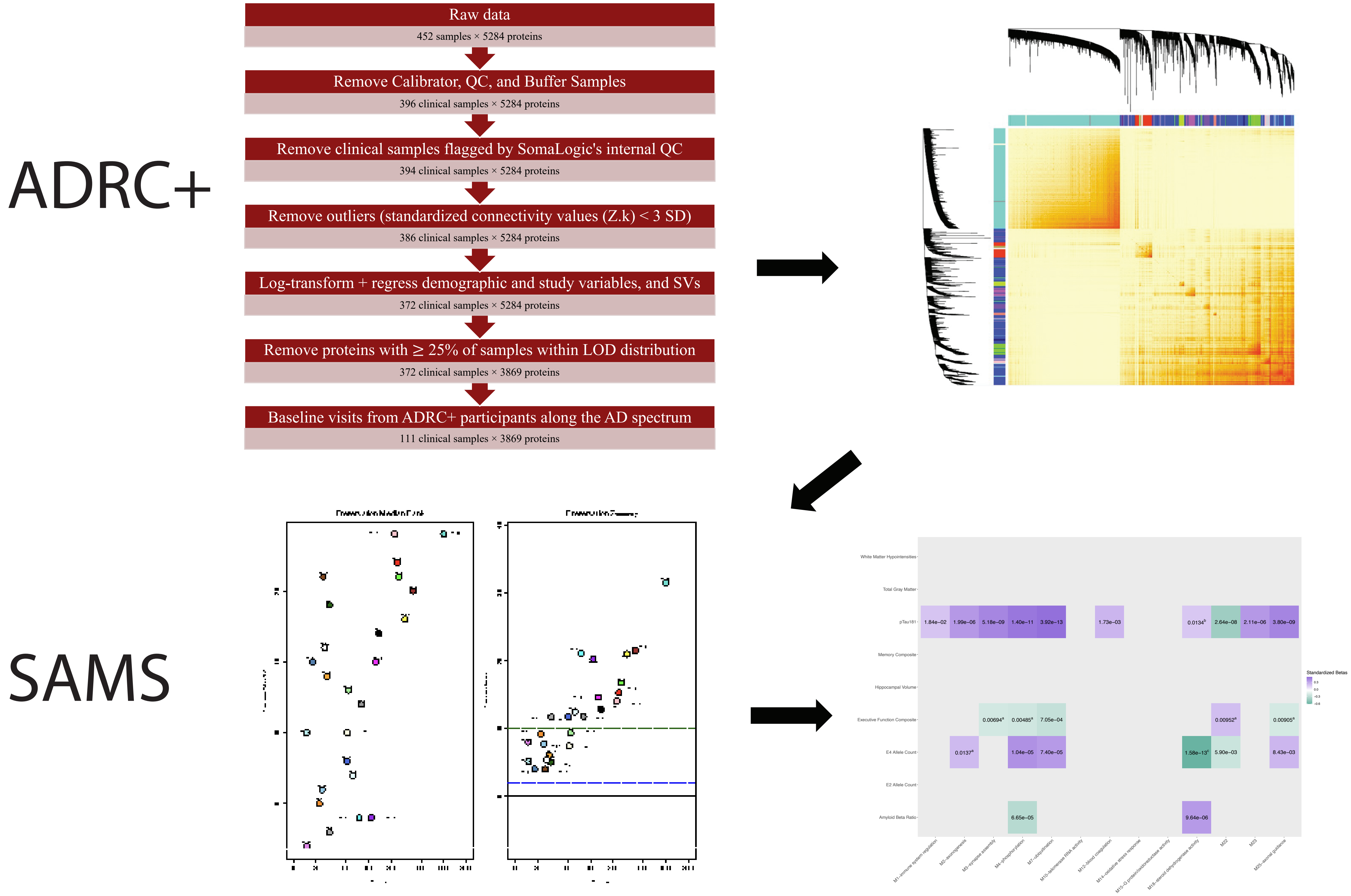

Supplemental Figure 1. Study overview figure. Steps taken to build a protein co-expression network (top) and analyze it among a cohort of clinically unimpaired individuals. For the ADRC+ cohort (left to right): data cleaning steps and sample exclusion criteria depicted in a flow chart, followed by network construction, depicted by a dendrogram, the input correlation matrix and module assignments. For the SAMS cohort (left to right): a module preservation analysis used to replicate the original cohort, followed by analysis of module/phenotype relationships.
