# Supplementary material for: Post‐translational modifications linked to preclinical Alzheimer's disease–related pathological and cognitive changes
Source: Alzheimers Dement. 2023 Dec 25;20(3):1851–67. doi: 10.1002/alz.13576 (PMC10984434; doi:10.1002/alz.13576)
Supplement: Supplementary file 3 — Supporting Information [file ALZ-20-1851-s008.pdf]

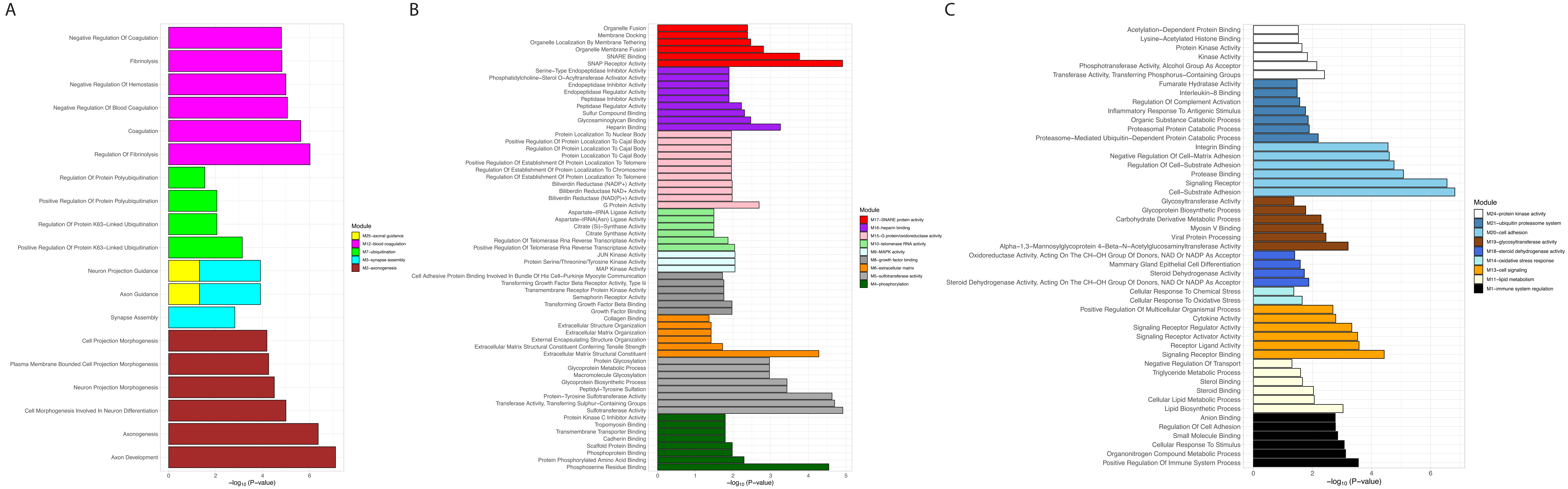


**Supplemental Figure 2. Gene ontology analysis for all modules.** Functional annotations derived from gene ontology analyses of all 25 co-expression network modules, conducted using g:Profiler. Only 22 contained functional enrichments that exceeded significance thresholds, and they are depicted here. The top six most significant gene ontology (GO) biological process and/or molecular function terms per module are displayed (y-axis), against their respective -log10 p-values (x-axis). A) Gene ontology results against a custom background of all SomaLogic-quantified proteins, adjusted using the default multiple comparison algorithm (g:SCS). B) Gene ontology results against a custom background of all SomaLogic-quantified proteins, adjusted using FDR correction. C) Gene ontology results against a background of all annotated genes, adjusted using the default multiple comparison algorithm (g:SCS).
