# Supplementary material for: Post‐translational modifications linked to preclinical Alzheimer's disease–related pathological and cognitive changes
Source: Alzheimers Dement. 2023 Dec 25;20(3):1851–67. doi: 10.1002/alz.13576 (PMC10984434; doi:10.1002/alz.13576)
Supplement: Supplementary file 4 — Supporting Information [file ALZ-20-1851-s004.pdf]

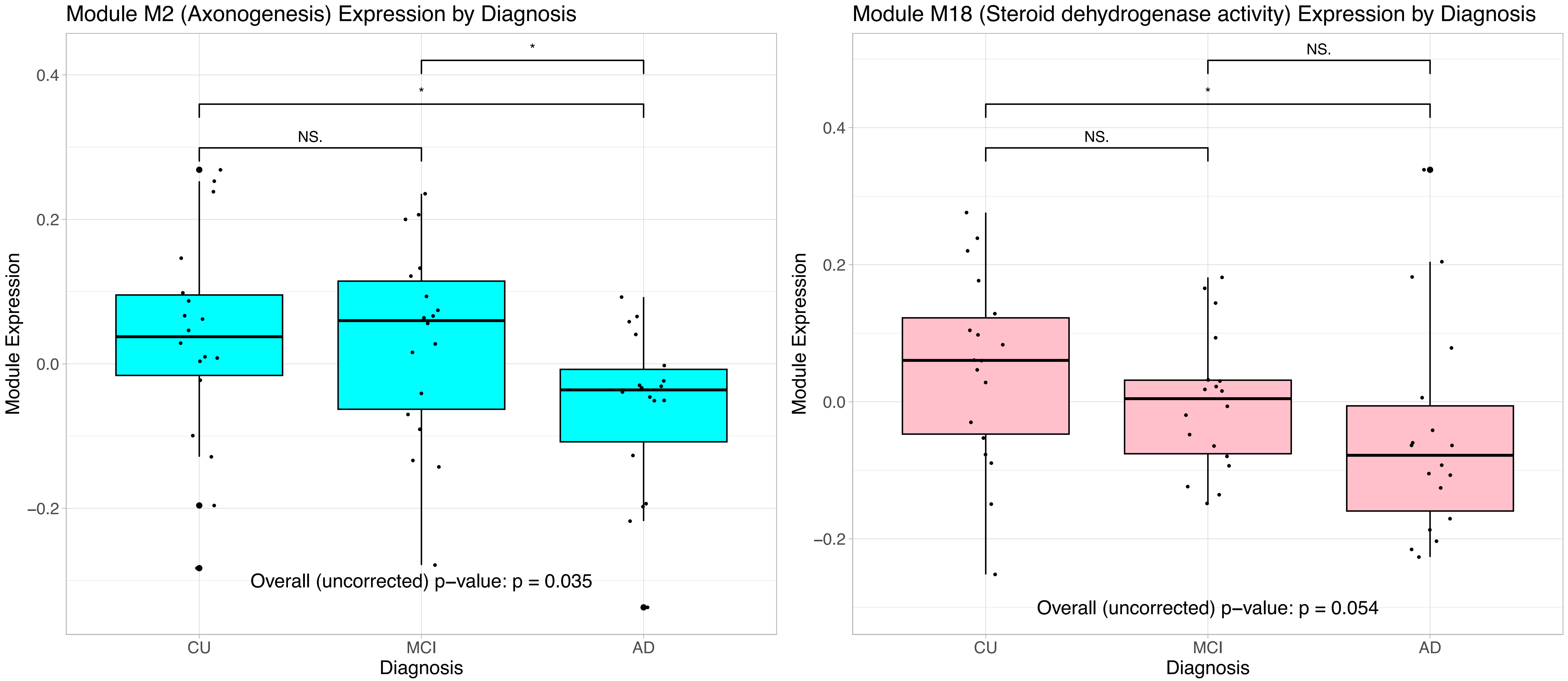

Supplemental Figure 3. Modules by clinical disease stage for relationships significant before multiple comparison correction. A box plot illustrating the results of Kruskall-Wallis tests for one-way ANOVA used to calculate module eigenprotein relationships to clinical disease stage. Modules whose significance did not survive FDR correction for multiple comparisons — on the left, module M2 (axonogenesis) and on the right, module M18 (steroid dehydrogenase activity) — are depicted here.
