# Supplementary material for: Post‐translational modifications linked to preclinical Alzheimer's disease–related pathological and cognitive changes
Source: Alzheimers Dement. 2023 Dec 25;20(3):1851–67. doi: 10.1002/alz.13576 (PMC10984434; doi:10.1002/alz.13576)
Supplement: Supplementary file 6 — Supporting Information [file ALZ-20-1851-s006.pdf]

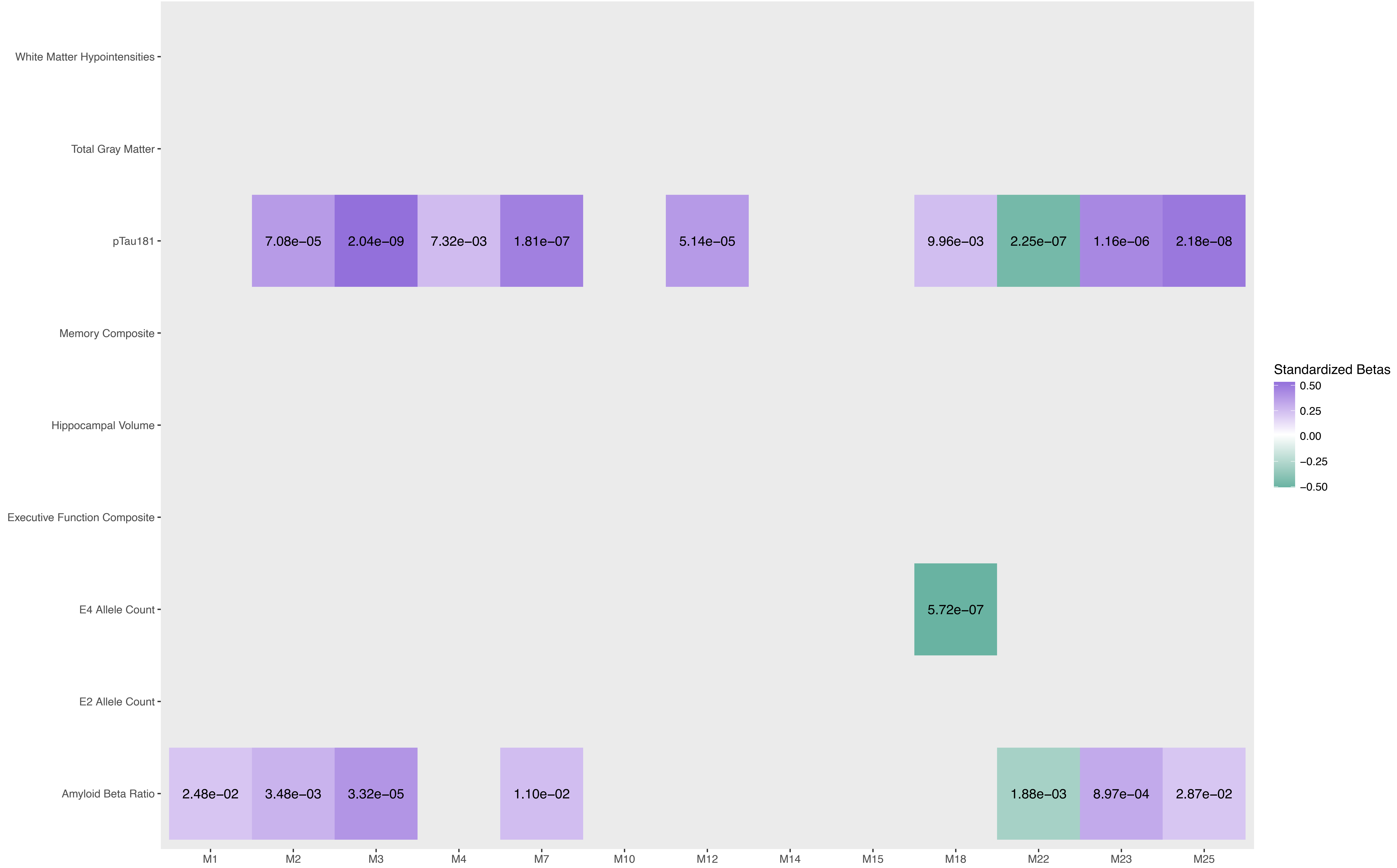

Supplemental Figure 5. Module/phenotype relationships among amyloid-negative participants within the SAMS cohort. Heat maps visualizing module relationships to cognition, AD pathology, genotype, and structural MRI outcomes among amyloid-negative (Aβ-) participants within the independent SAMS CU cohort. Only module/phenotype relationships significant aftermultiple comparison correction are depicted. Heatmap colors range from turquoise to purple, reflecting the magnitude and direction of standardized beta values. The text within heat map cells are the unadjusted p-values for each association.
