# Supplementary material for: Post‐translational modifications linked to preclinical Alzheimer's disease–related pathological and cognitive changes
Source: Alzheimers Dement. 2023 Dec 25;20(3):1851–67. doi: 10.1002/alz.13576 (PMC10984434; doi:10.1002/alz.13576)
Supplement: Supplementary file 5 — Supporting Information [file ALZ-20-1851-s005.pdf]

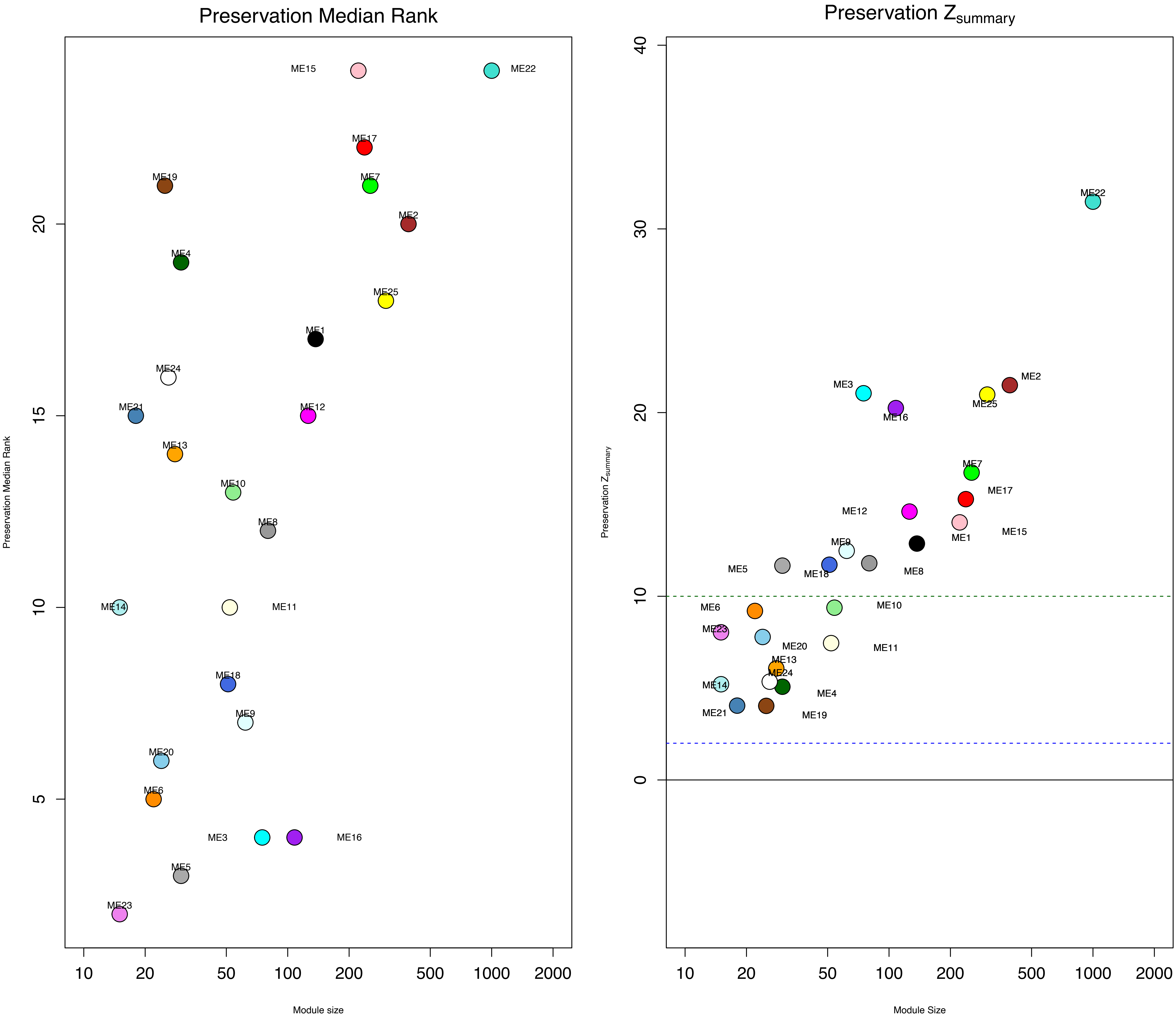

Supplemental Figure 4. Module preservation analysis within the independent SAMS cohort.
Depiction of module preservation analysis results, which quantify the extent to which protein co-expression network modules discovered in the ADRC+ cohort were replicated within the independent SAMS CU cohort. Using WGCNA, we calculated module preservation statistics that captured how well the strength of the interrelationships between nodes (module density), as well as connectivity patterns (module connectivity) across nodes, were preserved. On the left are module medianRank values, reflecting the relative rankings of modules across all preservation statistics (y-axis), plotted against module size (x-axis). On the right are module ZSummary values (y-axis) — i.e. the mean of the module density and connectivity categories of preservation statistics —, plotted against module size (x-axis). The green dotted line reflects the cutoff for highly preserved modules (!Summary > 10), whereas the blue dotted line reflects the minimum cutoff for module preservation, even if weak (2 < !Summary < 10).
