# Supplementary material for: Post‐translational modifications linked to preclinical Alzheimer's disease–related pathological and cognitive changes
Source: Alzheimers Dement. 2023 Dec 25;20(3):1851–67. doi: 10.1002/alz.13576 (PMC10984434; doi:10.1002/alz.13576)
Supplement: Supplementary file 8 — Supporting Information [file ALZ-20-1851-s001.pdf]

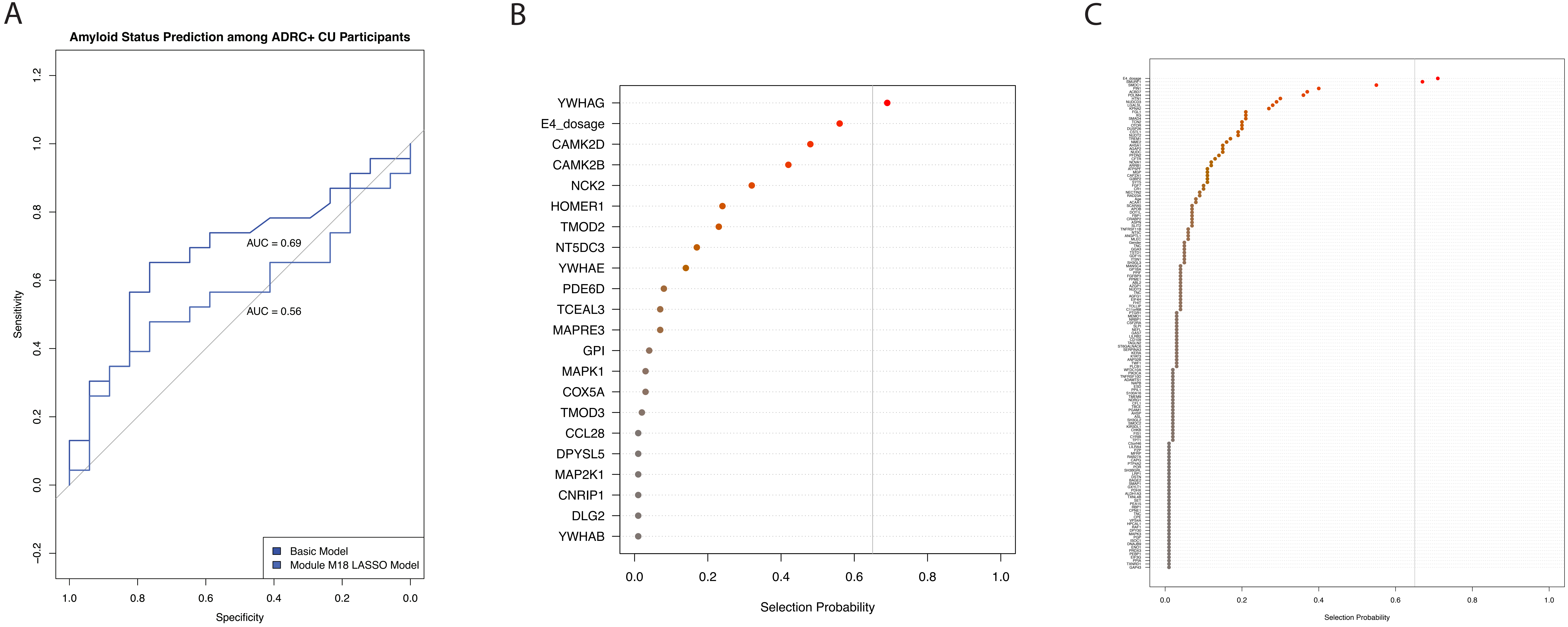

Supplemental Figure 7. Results from LASSO regression with stability selection. A) Receiver operator characteristic (ROC) curves depicting the classification performance (sensitivity vs. specificity) of models predicting amyloid status among a test set of CU ADRC+ participants. In blue are results from a basic logistic regression model including APOE ε4 allele count, sex, and age (AUC = 0.71). In royal blue are results from a LASSO regression model derived from M18-steroid dehydrogenase activity. B) Selection probabilities per model parameter for M4-phosphorylation, derived from performing LASSO regression across 50 different randomly-selected subsamples of data and ranking the features most likely to be selected from greatest to least. A probability threshold of 65% was used as a cutoff to identify stably selected model features. C) Selection probabilities per model parameter for M7-ubiquitination, derived from performing LASSO regression across 50 different randomly-selected subsamples of data and ranking the features most likely to be selected from greatest to least. A probability threshold of 65% was used as a cutoff to identify stably selected model features.
