# Supplementary material for: Post‐translational modifications linked to preclinical Alzheimer's disease–related pathological and cognitive changes
Source: Alzheimers Dement. 2023 Dec 25;20(3):1851–67. doi: 10.1002/alz.13576 (PMC10984434; doi:10.1002/alz.13576)
Supplement: Supplementary file 7 — Supporting Information [file ALZ-20-1851-s009.pdf]

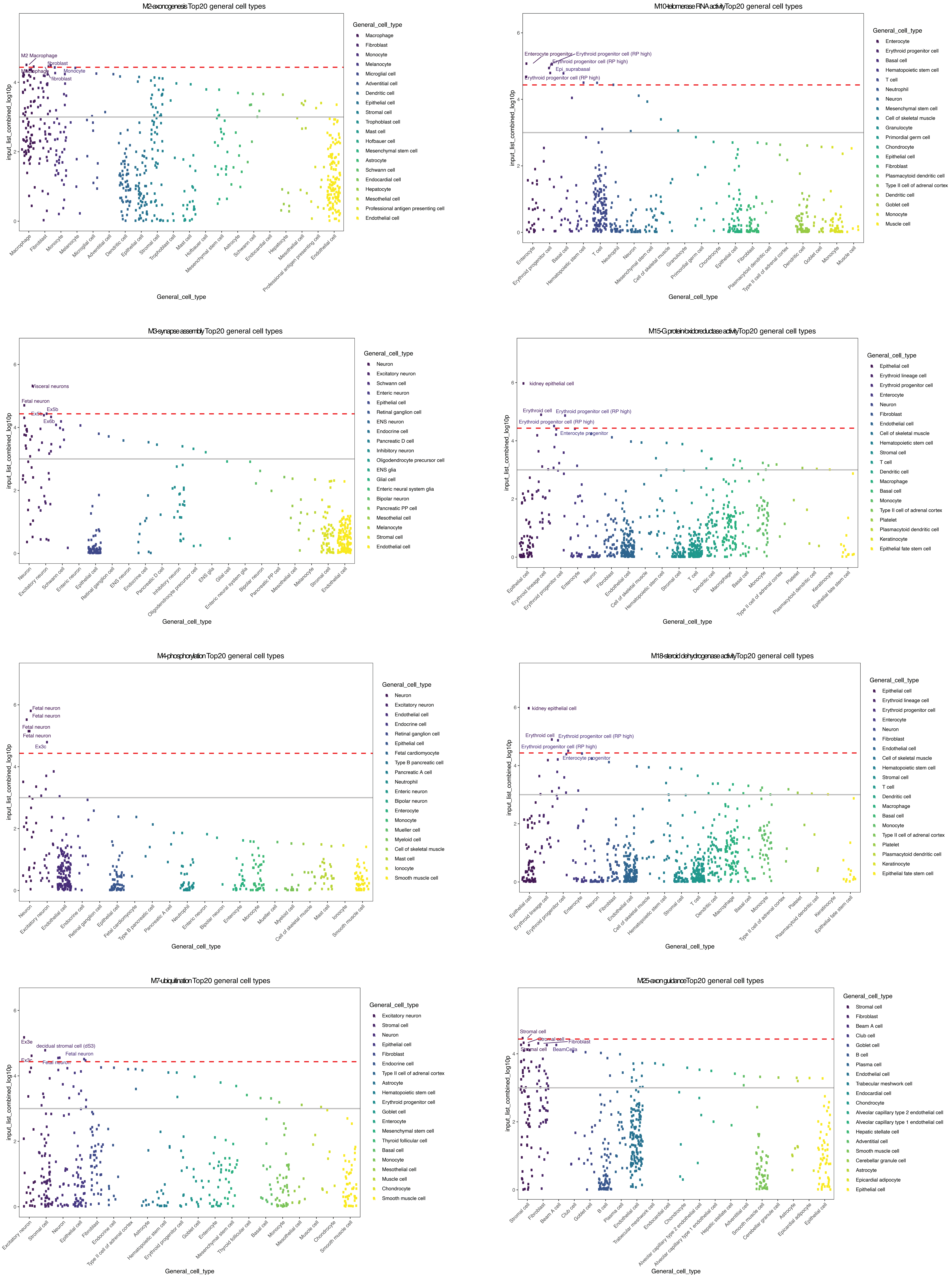

Supplemental Figure 6. Cell-type enrichment analysis. Depiction of general cell types enriched above a Bonferroni-corrected threshold combined *p*-value of 3.69 10-5. Enriched modules include (left, top to bottom) M2-axonogenesis, M3-synapse assembly, M4-phosphorylation, M7-ubiquitination (right, top to bottom) M10-telomerase RNA activity, M15-G protein/oxidoreductase activity, M18-steroid dehydrogenase activity, M25-axon guidance.
